# Community Health Worker and Mobile Health Interventions for Quality of Life Among Young Adults With Sickle Cell Disease: A Randomized Clinical Trial

**DOI:** 10.1001/jamanetworkopen.2025.43571

**Published:** 2025-11-17

**Authors:** Sophia Jan, Caren Steinway, Tanisha Belton, Justine Shults, Laura Bennett, Olivia Teng, Heather Griffis, Banu Aygun, Abena Appiah-Kubi, Nataly Apollonsky, Donna Boruchov, Omar Niss, Lisa Schwartz, Lori Crosby, Lamia Barakat, Biree Andemariam, Siddika S. Mulchan, David Rubin, Kim Smith-Whitley

**Affiliations:** 1 Department of Pediatrics, Northwell Health, Long Island, New York; 2 Department of Pediatrics, Cohen Children’s Medical Center, New Hyde Park, New York; 3 PolicyLab, Division of General Pediatrics, Children’s Hospital of Philadelphia, Philadelphia, Pennsylvania; 4 Children’s Hospital of Philadelphia, Philadelphia, Pennsylvania; 5 Department of Biostatistics, Epidemiology and Informatics. Perelman School of Medicine at University of Pennsylvania, Philadelphia; 6 Department of Biomedical and Health Informatics, Children’s Hospital of Philadelphia, Philadelphia, Pennsylvania; 7 Drexel University College of Medicine, Philadelphia, Pennsylvania; 8 St Christopher’s Hospital for Children, Philadelphia, Pennsylvania; 9 Connecticut Children’s Medical Center, Hartford; 10 Department of Pediatrics, Cincinnati Children’s Hospital Medical Center, Cincinnati, Ohio; 11 Division of Hematology, Cincinnati Children’s Hospital Medical Center, Cincinnati, Ohio; 12 Division of Oncology, Children’s Hospital of Philadelphia, Philadelphia, Pennsylvania; 13 Behavioral Medicine and Clinical Psychology, Cincinnati Children’s Hospital Medical Center, Cincinnati, Ohio; 14 New England Sickle Cell Institute, University of Connecticut Health, Farmington; 15 Department of Pediatrics, University of Connecticut School of Medicine, Farmington; 16 University of California Health, Oakland; 17 Division of General Pediatrics, Children’s Hospital of Philadelphia, Philadelphia, Pennsylvania

## Abstract

**Importance:**

Young adults with sickle cell disease (SCD) experience challenges transitioning from pediatric to adult care, leading to increased morbidity and mortality.

**Objective:**

To evaluate the effectiveness of community health worker (CHW) support or a mobile health application (mHealth) compared with enhanced usual care (EUC) in improving health-related quality of life (HRQOL) for young adults with SCD transitioning to adult care.

**Design, Setting, and Participants:**

The Community Health Workers and Mobile Health for Emerging Adults Transitioning Sickle Cell Disease Care study was an observer-blinded, multicenter, randomized clinical trial performed at 5 US children’s hospitals with a recruitment period from January 15, 2019, to December 31, 2022, and data analysis performed from September 30, 2024, to June 30, 2025. Participants were 17 years or older with SCD.

**Interventions:**

Participants were randomized 1:1:1 to 6 months of EUC, CHW plus EUC, or mHealth plus EUC. Both interventions included goal setting, self-management, skill development, symptom tracking, and transition support. The CHW plus EUC intervention provided weekly synchronous support primarily via phone calls, while mHealth plus EUC offered virtual peer support via interaction with discussion boards. The EUC group received standard care consisting of a transition checklist for consistency across sites.

**Main Outcomes and Measures:**

The main outcome was HRQOL, assessed using the Pediatric Quality of Life Inventory (PedsQL) for SCD module. Clinically meaningful improvement was prespecified as a 10-point change. Secondary outcomes included SCD knowledge, transition readiness, and social support. All outcomes were collected at baseline and follow-ups at 6, 12, and 18 months.

**Results:**

Of the 700 eligible patients across the 5 sites, 405 were enrolled, and 375 participants with SCD were randomized, 191 (51.5%) of whom were women. The mean (SD) age was 18.9 (1.9) years; the median age was 18.0 (IQR, 17-20) years. Baseline demographic data, clinical characteristics, and markers of disease severity were comparable across the study groups. At 6 months, the CHW plus EUC group showed modest improvements in HRQOL compared with the EUC group at 2.67 (95% CI, 0.25-5.09) at 6 months; there was no change for the mHealth plus EUC group at 0.73 (95% CI, −1.48 to 2.93) at 6 months; and the EUC group had a decline of 2.58 (95% CI, −4.67 to −0.49). CHW support demonstrated the greatest improvement in PedsQL scores compared with EUC at 6 (5.25 [95% CI, 2.05-8.45] points), 12 (5.56 [95% CI, 1.52-9.61] points), and 18 (6.14 [95% CI, 1.75-10.54] points) months. The mHealth plus EUC intervention demonstrated improvement in PedsQL scores at 6 months only (3.31 [95% CI, 0.27-6.35] points). Throughout the study, the HRQOL for the EUC group declined. No significant differences were found in secondary outcomes.

**Conclusions and Relevance:**

Although neither intervention met the prespecified 10-point threshold for a large clinical effect, the CHW intervention produced a significant and durable improvement in HRQOL that halted the decline observed in EUC. This sustained effect during the 18 months of follow-up suggests that CHW support provides a clinically relevant benefit for young adults with SCD during their transition to adult care. Integrating such programs into routine care could improve outcomes for this vulnerable population.

**Trial Registration:**

ClinicalTrials.gov Identifier: NCT03648710

## Key Points

**Question:**

What is the comparative effectiveness of a community health worker (CHW) program or a mobile health application (mHealth) on health-related quality of life (HRQOL) of young adults with sickle cell disease (SCD) transitioning from pediatric to adult care?

**Findings:**

In this randomized clinical trial of 375 young adults with SCD, HRQOL scores significantly improved between baseline and follow-up at 6, 12, and 18 months in the CHW and mHealth group compared with the enhanced usual care (EUC) group. The CHW plus EUC intervention showed the strongest improvement, and the mHealth plus EUC intervention demonstrated modest improvement at 6 months.

**Meaning:**

These findings suggest that CHW support and mHealth programs hold promise for enhancing HRQOL for young adults with SCD transitioning from pediatric to adult care.

## Teaser

This randomized clinical trial compares the effectiveness of community health worker (CHW) support and a mobile health application (mHealth) in improving health-related quality of life (HRQOL) and other relevant outcomes among young adults with sickle cell disease transitioning from pediatric to adult health care.

## Introduction

Sickle cell disease (SCD) is a genetic blood disorder in which sickle hemoglobin predominates and leads to red blood cell changes, hemolysis, and vaso-occlusion.^1^ Acute and chronic complications can increase in frequency as children age, and progressive end-organ damage is common.^2^ As early identification with newborn screening and medical treatment for SCD have significantly improved in the US during the last 30 years, most young adults with SCD are surviving to adulthood and must transition their health care from pediatric to adult-oriented medicine. However, barriers to optimal transition outcomes exist, limiting access to well-coordinated, high-quality care and contributing to worsening morbidity and a 7-fold increased mortality.^3^

Young adults with SCD are at risk of poor health-related quality of life (HRQOL) secondary to multiple medical and psychosocial factors.^3^ They have the highest rates of hospitalizations, emergency department visits, and hospital readmissions compared with other age groups. This increase in acute care utilization and mortality is partly due to difficulties in coordinating care during the transition to adult-oriented medicine.^4,5,6^

Moreover, the transition process occurs when young adults learn to assume greater responsibility for managing their health. Learning and engaging in self-management behaviors, which are daily, self-motivated, collaborative activities associated with symptom management, are key components of transition readiness and may improve transition outcomes. Inadequate care coordination, compounded with poor patient engagement and disease self-management skills, can increase the risk of neurologic complications.^5^

Despite these risks and associated negative health implications during the transition to adult care, few interventions targeting HRQOL in young adults with SCD have been evaluated. Two promising interventions are community health workers (CHWs) and mobile health applications (mHealth). CHW programs have demonstrated efficacy in improving self-management behaviors and health outcomes for young adults with other chronic conditions, particularly in areas with low resources.^6,7,8^ mHealth, including web-based and mobile text-messaging interventions, have demonstrated efficacy in improving self-management and adherence among young adults with chronic conditions.^9^

The objective of this study was to compare the effectiveness of CHW support and an mHealth application (app) in improving HRQOL and other relevant outcomes for young adults with SCD. We hypothesized that incorporating either a CHW or an mHealth app would improve HRQOL in young adults with SCD compared with to those receiving enhanced usual care (EUC) alone.

## Methods

### Study Design and Setting

This multicenter randomized clinical trial received lead Institutional Review Board approval from the Children’s Hospital of Philadelphia (CHOP), Philadelphia, Pennsylvania, followed by site-level approvals. This study was conducted in accordance with the Institutional Review Board–approved protocol given in Supplement 1. The study started recruiting January 15, 2019, and ended December 31, 2022. Reported findings were in accordance with the 2025 updated Consolidated Standards of Reporting Trials (CONSORT) guidelines. Eligible participants were 17 years or older with any SCD genotype, receiving care from a pediatric SCD center, and eligible for transition to adult care within 12 months. Exclusions consisted of no compatible mobile device access, severe intellectual disabilities precluding participation, and preferred language other than English. Participants were recruited from the 700 eligible patients with SCD receiving care at 1 of the following 5 pediatric SCD centers: CHOP; St Christopher’s Hospital for Children, Philadelphia, Pennsylvania; Cincinnati Children’s Hospital and Medical Center, Cincinnati, Ohio; Cohen Children’s Medical Center, New Hyde Park, New York; and Connecticut Children’s Medical Center, Hartford. Eligible patients were identified through reviews of each recruitment site’s daily hospitalized patients, weekly hematology appointments, and hematology practice registries. Systematic screening through the electronic medical record–based SCD registries and transition-related clinical decision support were also used. All patients provided written or oral informed consent.

Recruitment strategies included in-person enrollment during appointments or hospital admissions, mailed or emailed communication with recruitment letters and/or videos, and study flyers. Telephone recruitment and electronic consent were added to maintain recruitment goals during the COVID-19 pandemic. Retention strategies included follow-up procedures that were performed in person, over the telephone, or via email and/or text; automatic reminders; and incentives for effort.

Participants were randomized 1:1:1 to receive a 6-month intervention of EUC alone, CHW support plus EUC, or mHealth (iManage SCD app; Agency 39A) plus EUC. Randomization occurred after enrollment and completion of the baseline survey. Stratification according to recruitment site, age (≤21 or ≥22 years), and disease severity (mild or severe) was performed before assignment to a treatment group. Participants were asked to complete follow-up measures after the intervention period (at 6 months) and then again at 12 and 18 months.

Interventions were developed using the Social-Ecological Model for Adolescents and Young Adults Readiness to Transition model^9^ and the chronic care model,^10^ emphasizing enhanced knowledge, self-management, and social support.^11^ Further explanation and development of intervention groups can be found in the studies by Belton et al^12^ and Steinway et al.^11^

### Intervention Groups

#### EUC (Control)

EUC included the transition of care checklists (eAppendices 1 and 2 in Supplement 2) administered by social work teams or transition coordinators at each recruitment site. The elements of these checklists were based on existing checklists, including the Transfer of Care Checklist developed by the Got Transition–Center for Health Care Transition Improvement,^13^ and those developed by the National Institute for Child Health Quality.^14^ Elements of the checklist included the pediatrician meeting with the parent outside the examination room, a social work consult to screen and address sociodemographic risk factors, information on health insurance, identification of an adult hematologist and primary care clinician, a signed medical release, and a viewable medical record or a medical record sent to the physician. The development included a review of recruitment site investigators to help decide on the elements, based on transition elements and their institution.

#### CHW Plus EUC

In addition to EUC, the CHW plus EUC intervention provided weekly synchronous support to participants primarily via phone. Our framework was modeled using the IMPACT (Individualized Management for Patient-Centered Targets) program.^12,15,16,17^ Mirrored components included development of patient-centered goals and individualized action plans around self-care, symptom tracking, and transition to adult care; provision of information, skills, and tips; and tailored peer support.

All CHWs participated in a 2-week training session prior to being assigned study participants. These trainings covered local hospital and community knowledge, SCD and transition to adulthood education, an introduction to clinical research training, foundational CHW skills and concepts, and trial-specific aims and resources. In addition, CHWs met as a team during weekly check-ins and monthly huddles. Further description of the development process can be found in the study by Belton et al.^12^

#### mHealth Plus EUC

As with the CHW group, the mHealth group received EUC. The mHealth app was developed by Crosby et al^18^ in collaboration with young adults with SCD.^11^ The key elements included the development of patient-centered goals around self-care, symptom tracking, and the transition to adult care. The goal was to use virtual peer support to spread information, skills, and tips as well as to have a space where users could encourage others, form teams, and interact with fellow research participants with SCD. This was accompanied by daily symptom tracking and visual monitoring of app components for goal completion. Additionally, participants in the mHealth group received weekly SMS messages reminding them to engage with the mHealth app.

Virtual peer support was offered through interaction with discussion boards. Interface components (health behavior challenges, medication and symptom tracking, educational resources, discussion boards, and a medical summary), were tailored to support SCD management and monitoring by research staff.

The mHealth app was accessible via smartphones, tablets, or the web. The app has previously been shown to be highly feasible and beneficial for young adults with SCD.^18^ Application enhancements were identified through the collaboration of multistakeholder groups (ie, app development firm, clinicians, researchers, and young adults with SCD) across the 5 recruitment sites. Further descriptions of the enhanced app development process can be found in Steinway et al.^11^

### Data Collection and Outcome Measures

Data collection included patient-reported measures of HRQOL at baseline, at first follow-up (end of the 6-month intervention), second follow-up (12 months after baseline), and the final follow-up (18 months after baseline). Patients self-reported their gender identity (man, woman, other gender, and prefer not to answer), race (Black or African American, Native Hawaiian or Other Pacific Islander, White, multiracial, other [including American Indian or Alaska Native, Asian, and unspecified other race], and prefer not to answer), and ethnicity (Hispanic or non-Hispanic). The PedsQL SCD module is a specific instrument used in adolescents and young adults with chronic disease designed to measure HRQOL. This tool assesses different aspects of quality of life impacted by SCD using a Likert response scale (higher scores indicate better HRQOL and fewer SCD symptoms and problems). REDCap (Research Electronic Data Capture) was used to collect and manage study data.^19,20^

### Statistical Analysis

We evaluated the effectiveness of EUC vs CHW plus EUC support and mHealth plus EUC in improving HRQOL and other relevant outcomes in young adults with SCD. Descriptive statistics are presented as counts and percentages for categorical variables. The χ^2^ test or Fisher exact test for categorical variables and Kruskal-Wallis test for ordinal variables were used for initial comparisons of groups at baseline. The primary outcome focused on HRQOL using PedsQL SCD. We fitted a mixed-effects model with participant-level random intercepts to account for potential correlation among repeated measurements per participant. Time was modeled using cubic splines with knots at 0, 6, 12, and 18 months, the intended times of measurement. The longitudinal model also included indicator variables for each intervention group (CHW plus EUC and mHealth plus EUC) and time by intervention group interaction terms. No additional variables were included in the model because initial descriptive analyses failed to identify meaningful differences in demographic variables, clinical variables, or clinical markers of disease between intervention groups at baseline. We then used linear combinations of regression coefficients from the fitted model to obtain estimates of baseline and follow-up total PedsQL scores within groups as well as changes since baseline within groups and differences in changes between groups, with 95% CIs.

We hypothesized that participants in either intervention group (CHW or mHealth) would show greater improvement in PedsQL scores compared with participants in the EUC group. A clinically meaningful difference was prespecified as a 10-point change in PedsQL SCD Module Total Score. With our final sample of 375 participants, we had 80% power to detect an 8.3-point change in PedsQL SCD Module Total score and 4.7-point change in PedsQL 4.0 Generic Core Scales Young Adult scale. For longitudinal analysis, we used mixed-effects models with appropriate correlation structures for repeated measures. A key strength of the mixed-effects longitudinal model is that it uses all observed data, including from participants with incomplete follow-up, which increases statistical power and reduces potential bias from attrition.

To explore the potential influence of intervention engagement (dose) on HRQOL, exploratory analyses were conducted focusing on the 6-month follow-up data. For the CHW intervention, a categorical dose variable was created representing low, medium, or high engagement based on the total number of meaningful interactions (low, <10 interactions; medium, 10-24 interactions; high, ≥25 interactions). For the mHealth plus EUC intervention, dose was categorized based on participant interaction with the mobile app and/or SMS messages (low, no interaction with texts or app; medium, some interaction with texts or app but below high threshold; high, responding to >20 texts or interacting with the app >30 times).

Separate linear mixed-effects models were fitted for participants within each intervention group (CHW plus EUC and mHealth plus EUC) using PedsQL total scores at baseline and 6 months. For each group, a reduced model included elapsed time as the only explanatory variable. A full model was then fitted, adding the categorical dose level variable and an interaction term between dose and elapsed time. Model comparison was performed using the Akaike information criterion and the bayesian information criterion to assess whether the inclusion of dose terms significantly improved model fit.

Data were analyzed from September 30, 2024, to June 30, 2025. All analyses were performed using SAS Enterprise Guide, version 8.3 (SAS Institute Inc) and Stata, version 18 (StataCorp LLC). We used 2-sided tests of hypothesis and *P* < .05 as the criterion for statistical significance.

## Results

### Participants

**Figure 1.**
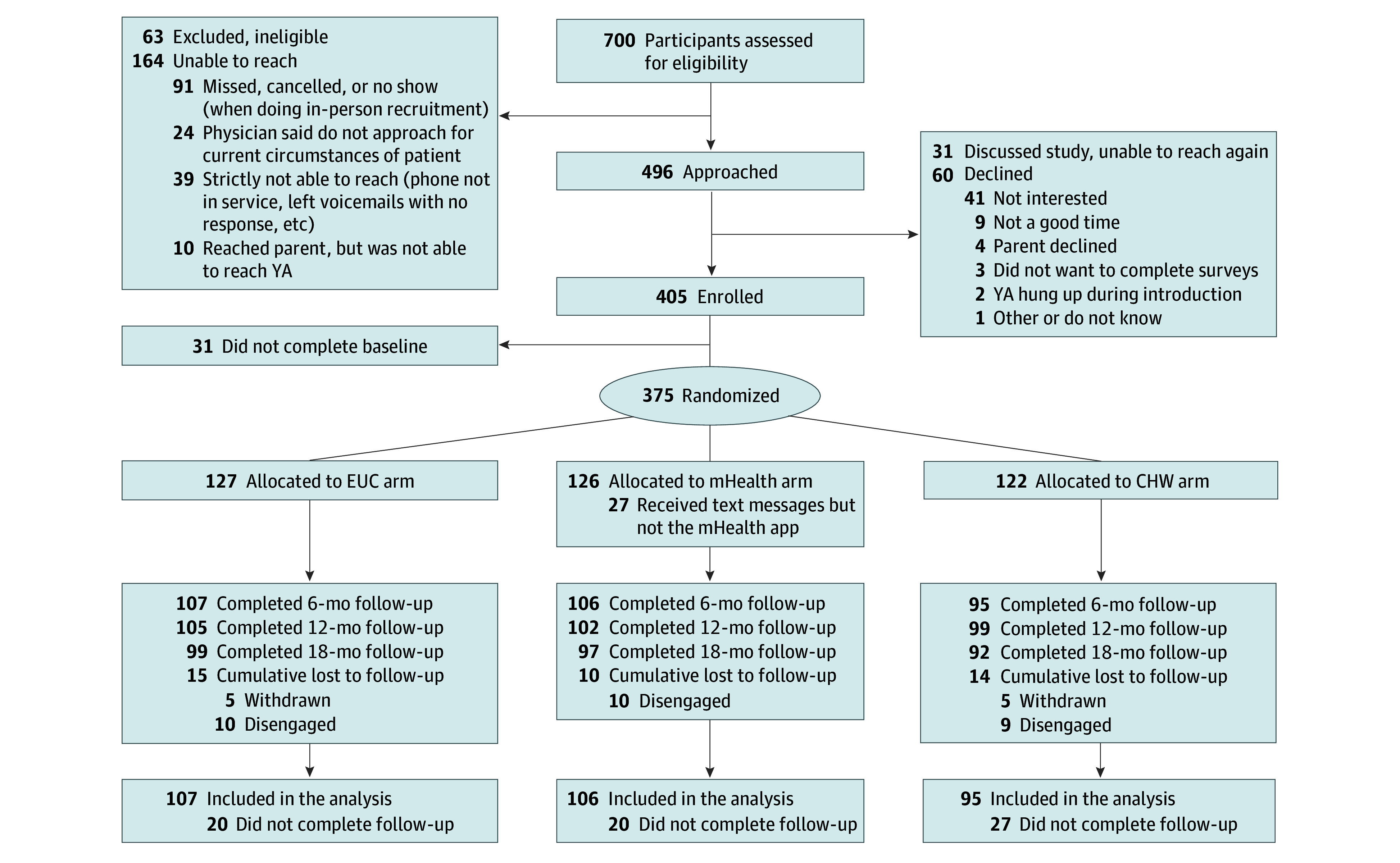
Study Flowchart As shown in the flowchart, the pattern of missing follow-up data was largely intermittent. Of the 67 participants with missing data at 6 months, 62 (92.5%) returned for a subsequent follow-up. Of the 69 participants with missing data at 12 months, 53 (76.8%) returned for the 18-month follow-up. CHW indicates community health workers; EUC, enhanced usual care; and mHealth, mobile health application.

Of the 700 eligible patients among the 5 sites, 405 were enrolled and a total of 375 participants were randomized during the recruitment period (January 15, 2019, to December 31, 2022). A total of 127 patients were allocated to the EUC group, 122 to the CHW plus EUC group, and 126 to the mHealth plus EUC group (Figure 1). Baseline demographic data, clinical characteristics, and markers of disease severity were comparable across the study groups (Table 1). Among participants, 337 (90.0%) were 17 to 21 years of age; the mean (SD) age was 18.9 (1.9); median age, 18 (IQR, 17-20) years. Of 371 participants with gender identity data available, 178 (48.0%) were men, 191 (51.5%) were women, 1 (0.3%) identified as other gender, and 1 (0.3%) preferred not to answer. Among 371 participants with race data available, 335 (90.3%) were Black or African American, 1 (0.3%) was Native Hawaiian or Other Pacific Islander, 2 (0.5%) were White, 15 (4.0%) were multiracial, 19 (4.0%) were of other race, and 3 (0.8%) preferred not to answer. Among 371 participants with ethnicity data available, 31 (8.4%) were Hispanic, 335 (90.3%) were non-Hispanic, and 5 (1.3%) preferred to not answer. Two hundred twenty-seven participants (60.5%) indicated having public or governmental insurance.

**Table 1.** Demographic and Clinical Characteristics of Patients at Baseline^a^

Characteristic	Study group, No./total No. (%)			
	Overall (N = 375)	EUC only (n = 127)	CHW plus EUC (n = 122)	mHealth plus EUC (n = 126)
Site				
Cohen Children’s Medical Center	91/375 (24.3)	32/127 (25.2)	28/122 (23.0)	31/126 (24.6)
Children’s Hospital of Philadelphia	143/375 (38.1)	46/127 (36.2)	49/122 (40.2)	48/126 (38.1)
Cincinnati Children’s Hospital	37/375 (9.9)	12/127 (9.4)	11/122 (9.0)	14/126 (11.1)
Connecticut Children’s Medical Center	43/375 (11.5)	15/127 (11.8)	14/122 (11.5)	14/126 (11.1)
St Christopher’s Hospital for Children	61/375 (16.3)	22/127 (17.3)	20/122 (16.4)	19/126 (15.1)
Age at baseline, y				
<17	4/375 (1.1)	0/127	4/122 (3.3)	0/126
17-21	337/375 (89.9)	116/127 (91.3)	106/122 (86.9)	115/126 (91.3)
≥22	34/375 (9.1)	11/127 (8.7)	12/122 (9.8)	11/126 (8.7)
Age at baseline, mean (SD), y	18.9 (1.9)	19.0 (1.9)	18.9 (2.1)	18.9 (1.8)
Gender identity				
Men	178/371 (48.0)	63/127 (49.6)	56/118 (47.5)	59/126 (46.8)
Women	191/371 (51.5)	62/127 (48.8)	62/118 (52.5)	67/126 (53.2)
Other	1/371 (0.3)	1/127 (0.8)	0/118	0/126
Prefer not to answer	1/371 (0.3)	1/127 (0.8)	0/118	0/126
Race				
Black or African American	335/371 (90.3)	119/127 (93.7)	106/118 (89.8)	110/126 (87.3)
Native Hawaiian or Other Pacific Islander	1/371 (0.3)	1/127 (0.8)	0/118	0/126
White	2/371 (0.5)	0/127	1/118 (0.8)	1/126 (0.8)
Multiracial	15/371 (4.0)	2/127 (1.6)	6/118 (5.1)	7/126 (5.6)
Other^b^	15/371 (4.0)	5/127 (3.9)	5/118 (4.2)	5/126 (4.0)
Prefer not to answer	3/371 (0.8)	0/127	0/118	3/126 (2.4)
Ethnicity				
Hispanic	31/371 (8.4)	11/127 (8.7)	9/122 (7.4)	11/126 (8.7)
Non-Hispanic	335/371 (90.3)	115/127 (90.6)	108/122 (88.5)	112/126 (88.9)
Primary insurance type				
Public or government	227/375 (60.5)	75/127 (59.1)	82/122 (67.2)	70/126 (55.6)
Private	137/375 (36.5)	45/127 (35.4)	39/122 (32.0)	53/126 (42.1)
Other	3/375 (0.8)	1/127 (0.8)	1/122 (0.8)	1/126 (0.8)
None	8/375 (2.1)	6/127 (4.7)	0/122	2/126 (1.6)
Currently using hydroxyurea	188/371 (50.7)	54/124 (43.5)	58/121 (47.9)	76/126 (60.3)
Currently receiving chronic transfusions	52/371 (14.0)	16/124 (12.9)	22/121 (18.2)	14/126 (11.1)
History of stroke	22/371 (5.9)	9/124 (7.3)	5/121 (4.1)	8/126 (6.3)
History of acute chest syndrome	144/371 (38.8)	53/124 (42.7)	39/121 (32.2)	52/126 (41.3)
No. of visits to the ED in the last 6 mo				
0	170/372 (45.7)	55/125 (44.0)	46/122 (37.7)	69/125 (55.2)
1	68/372 (18.3)	27/125 (21.6)	27/122 (22.1)	14/125 (11.2)
2	58/372 (16.0)	18/125 (14.4)	25/122 (20.5)	15/125 (12.0)
3	27/372 (7.3)	7/125 (5.6)	10/122 (8.2)	10/125 (8.0)
4	21/372 (5.6)	4/125 (3.2)	9/122 (7.4)	8/125 (6.4)
≥5	21/372 (5.6)	9/125 (7.2)	4/122 (3.3)	8/125 (6.4)
Prefer not to answer	7/372 (1.9)	5/125 (4.0)	1/122 (0.8)	1/125 (0.8)
No. of overnight hospital stays in the last 6 mo	372	125	122	125
0	199/372 (53.5)	66/125 (52.8)	59/122 (48.4)	74/125 (59.2)
1	74/372 (19.9)	24/125 (19.2)	31/122 (25.4)	19/125 (15.2)
2	37/372 (9.9)	14/125 (11.2)	16/122 (13.1)	7/125 (5.6)
3	24/372 (6.5)	5/125 (4.0)	9/122 (7.4)	10/125 (8.0)
4	32/372 (8.6)	12/125 (9.6)	6/122 (4.9)	14/125 (11.2)
Prefer not to answer	6/372 (1.6)	4/125 (3.2)	1/122 (0.8)	1/125 (0.8)

Abbreviations: CHW, community health worker; ED, emergency department; EUC, enhanced usual care; mHealth, mobile health application.

^a^ For certain characteristics, the total number of patients may not equal the number of patients in the overall cohort owing to unavailable data.

^b^ Includes American Indian or Alaska Native, Asian, and unspecified other race.

### Intervention Implementation

 Patient-reported measures of HRQOL were collected at a median 188 (IQR, 184-203) days for the first follow-up, 372 (IQR, 366-388) days for the second follow-up, and 555 (IQR, 550.0-568.0) days for the final follow-up. Almost all CHW plus EUC participants (111 of 122 [91.0%]) established contact, with a median of 25 (IQR, 21-30) contacts per participant during 6 months. Of 3114 total contacts, 1900 (61.0%) were meaningful interactions, with a mean (SD) of 21.0 (27.2) minutes each. Most interactions occurred via phone calls (1643 of 3021 [54.4%]) or text messages (1160 of 3021 [38.4%]).

For mHealth plus EUC, most participants (106 of 126 [84.1%]) interacted with either SMS messages or the app during the intervention period. However, sustained engagement with multiple app features was more limited (57 of 106 [53.8%]). Among engaged users, most joined at least 1 challenge (n = 42) and logged progress (758 events).

### Effect of Interventions on Overall PedsQL Scores

Despite varying engagement levels, both interventions showed improvements in HRQOL. Estimated scores for PedsQL showed differential changes across the 3 study groups during the 6-month period. At baseline, the PedsQL scores estimates from the mixed model were comparable across the groups: EUC at 56.8 (95% CI, 53.4-60.3), CHW plus EUC at 56.5 (95% CI, 53.0-60.0), and mHealth plus EUC at 56.0 (95% CI, 52.6-59.4). There was a notable decline in the EUC group to 54.2 (95% CI, 51.1-58.0), whereas the CHW plus EUC group improved to 59.3 (95% CI, 55.6-62.9), and the mHealth plus EUC group remained relatively stable at 56.8 (95% CI, 53.3-60.2). The estimated treatment effects compared with the EUC group at 180 days (estimated number of days to first follow-up) showed a larger improvement at 4.9 (95% CI, −0.1 to 9.8) in the CHW plus EUC group and no change in the mHealth plus EUC group (2.3 [95% CI, −2.5 to 7.1]) (Table 2). While these data points are a notable improvement, they are not clinically meaningful due to not having a difference of more than 10 points. No significant differences between groups were detected in secondary outcomes: SCD knowledge, transition readiness, or social support (eTable in Supplement 2).

**Table 2.** Estimates of Overall PedsQL From the Mixed Methods Model at Baseline by Number of Days to First Follow-Up

Estimation time by intervention	Overall PedsQL score estimate (95% CI)
**EUC (n = 131)**	
Baseline	56.8 (53.4-60.3)
180 d	54.2 (51.1-58.0)
365 d	54.5 (50.9-58.1)
540 d	53.5 (49.7-57.3)
**CHW plus EUC (n = 94)**	
Baseline	56.5 (53.0-60.0)
180 d	59.3 (55.6-62.9)
365 d	59.8 (56.0-63.6)
540 d	59.3 (55.3-63.4)
**mHealth plus EUC (n = 81)**	
Baseline	56.0 (52.6-59.4)
180 d	56.8 (53.3-60.2)
365 d	56.0 (52.4-59.6)
540 d	55.3 (51.4-59.1)

Abbreviations: CHW, community health worker; EUC, enhanced usual care; mHealth, mobile health application; PedsQL, Pediatric Quality of Life Inventory for sickle cell disease module.

**Figure 2.**
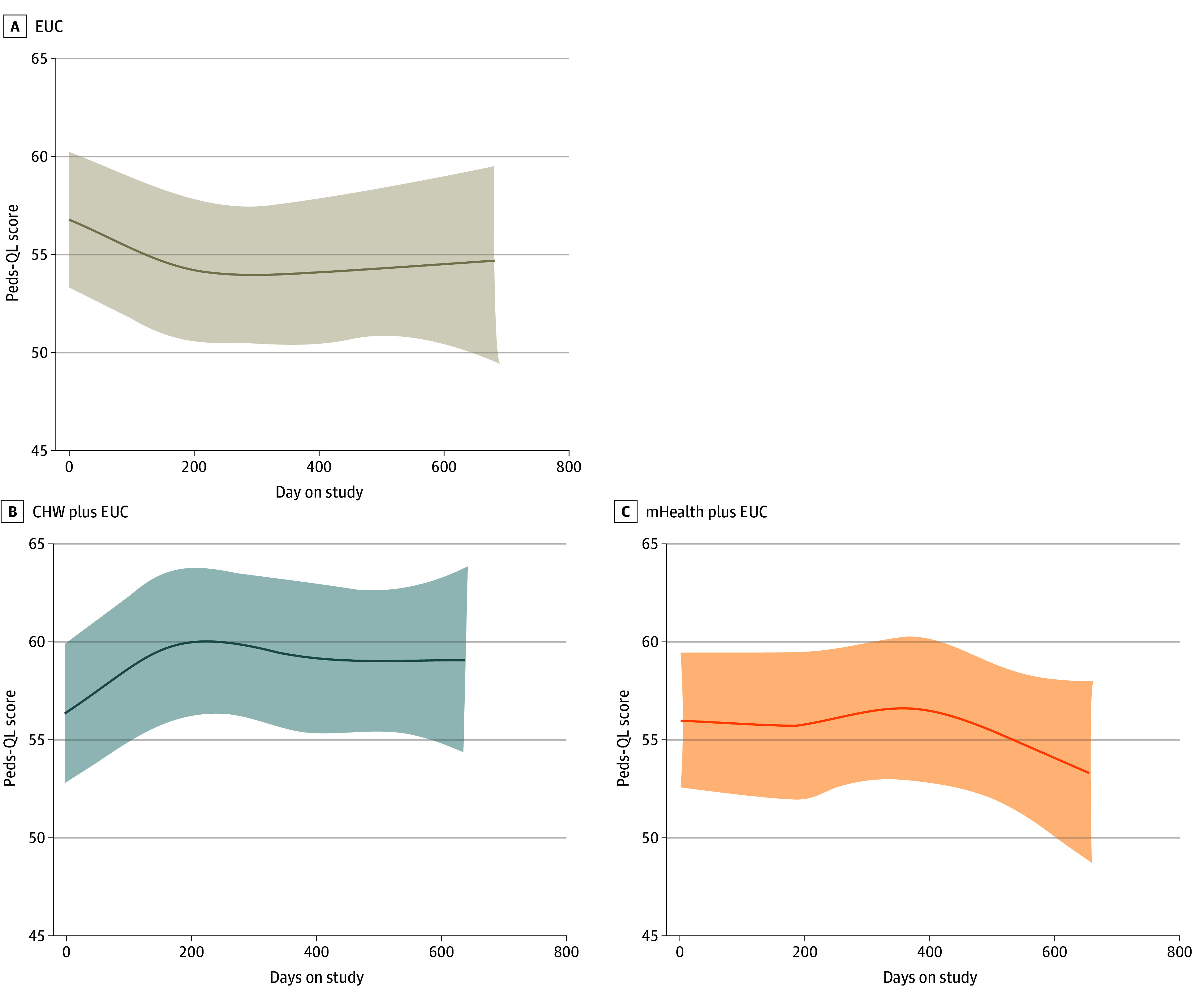
Fitted Values for Total Pediatric Quality of Life Inventory (PedsQL) for Sickle Cell Disease Module Results are shown as cubic spline regression over elapsed days. Shaded areas indicate 95% CIs. CHW indicates community health workers; EUC, enhanced usual care; and mHealth, mobile health application.

Throughout the study period, the PedsQL scores for both interventions increased from baseline to second follow-up (12 months after baseline), with slight dips at the third follow-up (18 months after baseline). We observed a significant overall change in the CHW plus EUC group (2.67 [95% CI, 0.25-5.09] at 6 months, 3.25 [95% CI, 0.29-6.22] at 12 months, and 2.80 [95% CI,-0.50-6.09] at 18 months), as shown in Table 3. A notable decrease during the 18-month period was observed for the EUC group (−2.58 [95% CI, −4.67 to −0.49] at 6 months, −2.31 [95% CI, −5.06 to 0.44] at 12 months, and −3.35 [95% CI, −6.26 to −0.43] at 18 months). The longitudinal patterns are shown in Figure 2. Statistically significant differences in change since baseline were identified between the 2 intervention arms and the EUC group. Significant differences in changes since baseline were identified between the CHW plus EUC group and the EUC group at 6 (5.25 [95% CI, 2.05-8.45] points), 12 (5.56 [95% CI, 1.52-9.61] points), and 18 (6.14 [95% CI, 1.75-10.54] points) months. Significant differences in changes since baseline were identified between the mHealth plus EUC group and the EUC group only at the 6-month follow-up (3.31 [95% CI, 0.27-6.35] points) but not the 12-month (2.47 [95% CI, −1.42 to 6.36] points) and 18-month (2.77 [95% CI, −1.44 to 6.98] points) follow-ups. The CHW plus EUC intervention showed the strongest improvement (from baseline) in PedsQL scores at 12 months (3.25 [95% CI, 0.29-6.22] points). The mHealth plus EUC intervention demonstrated modest improvement at 6 months (3.31 [95% CI, 0.27 to 6.35] points). However, the effects were not sustained after intervention completion, with 12- and 18-month scores at 2.47 points (95% CI, −1.42 to 6.36) and 2.77 points (95% CI, −1.44 to 6.98) points, respectively.

**Table 3.** PedsQL Changes From Baseline and Differences From EUC^a^

Measurement time for intervention	Overall PedsQL score change, median (IQR)	PedsQL change from baseline vs EUC (IQR)
**EUC (n = 107)**		
Baseline to first follow-up (6 mo)	−2.58 (−4.67 to −0.49)	NA
Baseline to second follow-up (12 mo)	−2.31 (−5.06 to 0.44)	NA
Baseline to third follow-up (18 mo)	−3.35 (−6.26 to −0.43)	NA
**CHW plus EUC (n = 94)**		
Baseline to first follow-up (6 mo)	2.67 (0.25 to 5.09)	5.25 (2.05 to 8.45)
Baseline to second follow-up (12 mo)	3.25 (0.29 to 6.22)	5.56 (1.52 to 9.61)
Baseline to third follow-up (18 mo)	2.80 (−0.50 to 6.09)	6.14 (1.75 to 10.54)
**mHealth plus EUC (n = 104)**		
Baseline to first follow-up (6 mo)	0.73 (−1.48 to 2.93)	3.31 (0.27 to 6.35)
Baseline to second follow-up (12 mo)	0.16 (−2.59 to 2.91)	2.47 (−1.42 to 6.36)
Baseline to third follow-up (18 mo)	−0.58 (−3.62 to 2.46)	2.77 (−1.44 to 6.98)

Abbreviations: CHW, community health worker; EUC, enhanced usual care; mHealth, mobile health application; NA, not applicable; PedsQL, Pediatric Quality of Life Inventory for sickle cell disease module.

^a^ Obtained from fitted values from cubic spline for each randomization group at elapsed days 0 (baseline), 180 (6 months), 365 (12 months), and 540 (18 months).

### Exploratory Dose-Effect Analysis

Exploratory analyses were conducted to assess whether the level of engagement (dose) with the CHW plus EUC or mHealth plus EUC interventions was associated with HRQOL outcomes at the 6-month follow-up. For both the CHW plus EUC and mHealth plus EUC interventions, the inclusion of dose level and dose-by-time interaction terms in the linear mixed-effects models did not result in statistically significant improvements in HRQOL. Furthermore, model comparison using the Akaike information criterion and the bayesian information criterion indicated that the reduced models (without dose-effect terms) were preferred for both interventions, suggesting that a statistically significant dose-response relationship was not detected within the parameters of this exploratory analysis.

### Acute Care Utilization as a Proxy for Disease Course

At baseline, the number of emergency department visits and hospitalizations in the preceding 6 months were comparable across the 3 groups (Table 1). To understand whether changes in disease course might have confounded HRQOL outcomes, acute care utilization was examined. Descriptively, at the 6-month follow-up (end of intervention), the overall pattern of health care utilization within the cohort largely reflected baseline status: low utilization at baseline generally remained low, and high utilization at baseline generally remained high, with no significant shifts in these patterns observed across the entire cohort at this time point. This suggests that the observed HRQOL differences between intervention groups at 6 months were not primarily driven by concurrent, large-scale changes in disease severity as proxied by acute care utilization. During the longer term (12 and 18 months), descriptive analysis of the overall cohort indicated that individuals who had high utilization at baseline showed some trend toward lower utilization at these later follow-ups.

## Discussion

This randomized clinical trial demonstrates that both CHW plus EUC support and mHealth plus EUC can enhance HRQOL for young adults with SCD transitioning to adult care. While neither intervention achieved clinically significant improvements in HRQOL (defined as 10-point change in PedsQL scores), the positive patterns and sustained effects during the 18-month study suggest promise for these approaches.

This study has 2 important findings. First, both CHW plus EUC and mHealth plus EUC interventions improved PedsQL scores among young adults with SCD during the 6-month intervention period. The CHW plus EUC intervention showed the strongest improvement (from baseline) in PedsQL scores at 12 months (3.25 [95% CI, 0.29-6.22] points). The CHW intervention also maintained and increased benefits through extended follow-up. At 12 and 18 months, PedsQL scores increased 5.56 (95% CI, 1.52-9.61) points and 6.14 (95% CI, 1.75-10.54) points, respectively, highlighting the potential benefits of personalized care and attention from CHWs. This significant and sustained improvement, while not meeting the 10-point threshold for clinically meaningful improvement, meets or exceeds the established minimal clinically important difference in PedsQL in other serious pediatric chronic conditions.^21,22,23^ The mHealth plus EUC intervention demonstrated a modest improvement at 6 months (3.31 [95% CI, 0.27 to 6.35] points). However, the effects were not sustained after intervention completion, with 12- and 18-month scores at 2.47 (95% CI, −1.42 to 6.36) and 2.77 (95% CI, −1.44 to 6.98) points, respectively. The control group experienced a decline in PedsQL scores, suggesting the need for future research into the long-term trajectory of HRQOL under EUC conditions and the sustained effects of interventions.

Our study reinforces the potential of CHWs and mHealth apps to enhance HRQOL, contributing valuable evidence to the current literature on health interventions. The scalability of CHWs and mHealth apps is particularly relevant for future policy development and implementation. This study’s findings regarding the positive impact of these interventions on overall quality of life, even though not clinically significant, further support the growing body of literature advocating for their integration into health care systems. Importantly, we also demonstrated that a CHW workforce can not only be targeted to focus on young adults with SCD, but also sustainably staffed by young adults with SCD.^12^

Additionally, our findings align with the growing recognition of the clinical significance of CHW programs and the momentum toward investing in them by health systems and professional organizations. This study contributes to the current literature^24,25^ by highlighting the positive impact of CHWs. The growing recognition for sustainable CHW programs from professional organizations and health care systems is evident in initiatives such as CHW pipeline training^26^ and commitments to seeking financial support.^21,22,26,27,28,29,30^

Similarly, mHealth apps are growing in recognition as an innovative tool for disease management, with notable applications in diabetes care.^23^ Ongoing developments in mHealth technology include symptom tracking and integration with electronic medical records,^31,32^ offering real-time data to health care clinicians and potentially reducing unplanned visits, conserving resources, and improving patient care management.

Importantly, these interventions are not mutually exclusive. Their combined use may offer a more comprehensive approach, addressing a broader range of challenges faced by patients with SCD during transition periods. CHWs provide personalized support, while mHealth apps may offer real-time care and potentially reduce costs. We advocate for future research to explore the synergistic benefits of these interventions and their combined potential to inform policy interventions.

### Strengths and Limitations

A key strength of this study is its focus on young adults with SCD. This population navigates a demanding transitional period, often juggling significant life changes such as entering the workforce or pursuing higher education, alongside fragmented health care experiences across multiple health systems and insurance networks. These challenges can have a negative impact on treatment adherence and engagement with SCD programs.^29,30^ Therefore, we chose to codevelop our interventions with young adults with SCD and other key stakeholders to create accessible interventions that not only effectively bridge disparate health care systems but are engaging and appealing.

Despite these strengths, our study has some limitations. Engagement varied significantly across intervention groups, highlighting implementation challenges that warrant attention in future work. Despite young adults’ participation in the development of the mHealth app, overall engagement within the app itself was relatively low. One reason may be that there were relatively few participants in the app at one time, contributing to fewer opportunities for engagement across users.^11^ Another limitation was that participants were recruited and enrolled in the study before and during the COVID-19 pandemic; there were important shifts in perspectives about health care, health care access, and disruptions in activities typically associated with transition to adulthood during this time.

## Conclusions

In this randomized clinical trial, neither CHW support nor the mHealth program met the prespecified 10-point threshold for a clinically meaningful improvement in HRQOL. However, the interpretation of this finding requires nuance, as the CHW intervention produced a statistically significant and sustained improvement during the study period. Furthermore, these interventions successfully reversed the negative HRQOL trajectory observed in the EUC group, a critical achievement during the vulnerable transition to adult care. These findings suggest that structured CHW support provides a durable, clinically relevant benefit for young adults with SCD. Future research should focus on optimizing and scaling these interventions to maximize their impact on patient well-being.

## Notes

Supplement 1.
Trial Protocol

Supplement 2. **eTable.** Change in Outcome Scores Between Baseline and First Follow-Up by Randomization Group **eAppendix 1.** Enhanced Usual Care Checklist (12 Months) **eAppendix 2.** Enhanced Usual Care Checklist (Social Work)

Supplement 3.
Data Sharing Statement
