# Supplementary material for: Community Health Worker and Mobile Health Interventions for Quality of Life Among Young Adults With Sickle Cell Disease: A Randomized Clinical Trial
Source: JAMA Netw Open. 2025 Nov 17;8(11):e2543571. doi: 10.1001/jamanetworkopen.2025.43571 (PMC12625686; doi:10.1001/jamanetworkopen.2025.43571)
Supplement: Supplement 1. — Trial Protocol [file jamanetwopen-e2543571-s001.pdf]

Title: **Management of Care Transitions for Emerging Adults with Sickle Cell Disease**

Short Title COMETS Trial

eIRB Number 18-015106

Protocol Date: December 15, 2020

Amendment 1 Date: July 3, 2018

Amendment 2 Date: September 24, 2018

Amendment 3 Date: November 20, 2018

Amendment 4 Date: March 12, 2019

Amendment 5 Date: October 21, 2019

Amendment 6 Date: January 2, 2020

Amendment 7 Date: March 12, 2020

Amendment 8 Date: August 4, 2020

Amendment 9 Date: August 28, 2020

Amendment 10 Date: December 15, 2020

Dr. David Rubin
2716 South Street Floor 10
Philadelphia, Pa 19126
Phone 215-590-3815
Email: rubin@email.chop.edu

Dr. Kim Smith- Whitley
3501 Civic Center Blvd
Philadelphia, PA 19104
Phone: 215-590-1662
Email: whitleyk@email.chop.edu

TABLE OF CONTENTS

Table of Contents ii
Abbreviations and Definitions of Terms iv
Abstract v
Table 1: Schedule of Study Procedures vii
Figure 1: Study Diagram viii

1 BACKGROUND INFORMATION AND RATIONALE 1

1.1 INTRODUCTION 1
1.2 NAME AND DESCRIPTION OF INVESTIGATIONAL PRODUCT OR INTERVENTION 1
1.3 RELEVANT LITERATURE AND DATA 5
1.4 COMPLIANCE STATEMENT 8

2 STUDY OBJECTIVES 8

2.1 PRIMARY OBJECTIVE (OR AIM) 8
2.2 SECONDARY OBJECTIVES (OR AIM) 8

3 INVESTIGATIONAL PLAN 9

3.1 GENERAL SCHEMA OF STUDY DESIGN 9
3.1.1 Screening Phase 9
3.1.2 Study Treatment Phase (start of the study intervention) 9
3.1.3 Follow-ups 9
3.2 ALLOCATION TO TREATMENT GROUPS AND BLINDING 9
3.3 STUDY DURATION, ENROLLMENT AND NUMBER OF SITES 10
3.3.1 Duration of Study Participation 10
3.3.2 Total Number of Study Sites/Total Number of Subjects Projected 10
3.4 STUDY POPULATION 10
3.4.1 Inclusion Criteria 10
1) Males or females age 17 years or older. 10
2) Have sickle cell disease, defined as those individuals with HbSS, HbSC, HbSβ^0Thal, HbSβ^+Thal genotypes 10
3) Receive care at a participating pediatric sickle cell disease center. 10
4) Appropriate for transfer to an adult hematologist within 12 months 10
3.4.2 Exclusion Criteria 10

4 STUDY PROCEDURES 11

4.1 SCREENING VISIT 11
4.2 STUDY TREATMENT PHASE 11
4.2.1 Visit 1 11
4.2.2 Visit 2 12
4.2.3 Visit 3 12
4.2.4 Visit 4: End of Study 12
4.3 SUBJECT COMPLETION/WITHDRAWAL. 13

5 STUDY EVALUATIONS AND MEASUREMENTS 14

5.1 SCREENING AND MONITORING EVALUATIONS AND MEASUREMENTS 14
5.1.1 Medical Record Review 14
5.2 EFFICACY EVALUATIONS 15
5.2.1 Diagnostic Tests, Scales, Measures, etc. 15

STATISTICAL CONSIDERATIONS 18

5.3 PRIMARY ENDPOINT 18
5.4 SECONDARY ENDPOINTS 18
5.5 STATISTICAL METHODS 18

5.5.1 Baseline Data ... *18*
5.5.2 Efficacy Analysis ... *18*
5.5.3 Safety Analysis ... *21*
5.6 SAMPLE SIZE AND POWER ... 21

6 SAFETY MANAGEMENT ... 23

6.1 CLINICAL ADVERSE EVENTS ... 23
6.2 ADVERSE EVENT REPORTING ... 23

7 STUDY ADMINISTRATION ... 23

7.1 TREATMENT ASSIGNMENT METHODS ... 23
7.1.1 Randomization ... *23*
7.1.2 Blinding ... *23*
7.2 DATA COLLECTION AND MANAGEMENT ... 23
7.3 CONFIDENTIALITY ... 24
7.4 REGULATORY AND ETHICAL CONSIDERATIONS ... 24
7.4.1 Data and Safety Monitoring Plan ... *24*
7.4.2 Risk Assessment ... *24*
7.4.5 Risk-Benefit Assessment ... *27*
7.5 RECRUITMENT STRATEGY ... 27
7.6 INFORMED CONSENT/ASSENT AND HIPAA AUTHORIZATION ... 28
7.7 PAYMENT TO SUBJECTS/FAMILIES ... 29
7.7.1 Payments to subject for time, effort and inconvenience (i.e. compensation) ... *29*

8 PUBLICATION ... 30

9 REFERENCES ... 31

ABBREVIATIONS AND DEFINITIONS OF TERMS

AE	Adverse event
SCD	Sickle cell disease
CHW	Community health worker
RCT	Randomized controlled trial
ASQ-Me	Adult Sickle Cell Quality of Life Measurement Information System
EMR	Electronic medical record

ABSTRACT

Context: (Background)

This staggering increase in mortality and acute care utilization during the transition period of adults with sickle cell disease (SCD) is partly due to difficulty coordinating care during the transition to adult care. Self-management support is a key component of the Chronic Care Model. The effectiveness of mHealth and tailored texting among emerging adults with SCD is still unknown. Furthermore, also unknown is the comparative effectiveness of CHW programs and mHealth.

Objectives: (primary and important secondary objectives)

Primary

- To compare the effectiveness of two self-management support interventions (community health workers and mobile health) versus enhanced usual care to improve health-related quality of life and acute care use for transitioning youth with SCD.

Secondary

- Identify and quantify whether patient activation, self-management behaviors, biologic markers, and transfer to adult care are mediators of intervention treatment effects
- Identify individual and family factors that moderate intervention treatment effects
- Explore the association of enhancements to usual care on pediatric and adult acute utilization

Study Design:

Multicenter, three-arm, open-label randomized controlled trial (RCT)

Setting/Participants:

The study will be conducted at multiple investigative sites in the United States. We estimate that there are approximately 745 eligible patients across the recruitment sites over the recruitment period. It is expected that approximately 60% will be enrolled to produce 450 evaluable subjects.

Inclusion Criteria

1) Males or females age 17 years or older
2) Have sickle cell disease, defined as those individuals with HbSS, HbSC, HbSβ^0Thal, HbSβ^+Thal , HbS-Other genotypes
3) Receive care at a participating pediatric sickle cell disease center.
4) Appropriate for transfer to an adult hematologist within 12 months

Exclusion Criteria

Individuals with an intellectual disability that is severe enough that the individual would not have the capacity to interact with a mobile or web-based program even with assistance or

have a conversation with a community health worker (i.e. non-verbal). Individuals without access to a mobile device, tablet or computer.

Study Interventions and Measures:

Control Arm: Enhanced Usual Care
Intervention One: Community Health Worker
Intervention Two: Mobile Health

Health-Related Quality of Life and acute care use for transitioning youth with SCD is the primary outcome of this study. They will be assessed by comparing the PEDsQL SCD and ASCQ-Me scores from the baseline visit to each follow-up visit.

TABLE 1: SCHEDULE OF STUDY PROCEDURES

**Study Phase**	**Screening**	**Intervention**	**Follow-up**		
**Visit Number**		**1**	**2**	**3**	**4**
**Study Days**					
Informed Consent/Assent	X				
Review Inclusion/Exclusion Criteria	X				
Demographics/Medical History	X				
SCD Disease Severity		X	X	X	X
Medical Chart Review	X		X	X	X
Randomization		X			
Baseline Survey Administered		X			
Follow-up Survey Administered			X	X	X

.

FIGURE 1: STUDY DIAGRAM

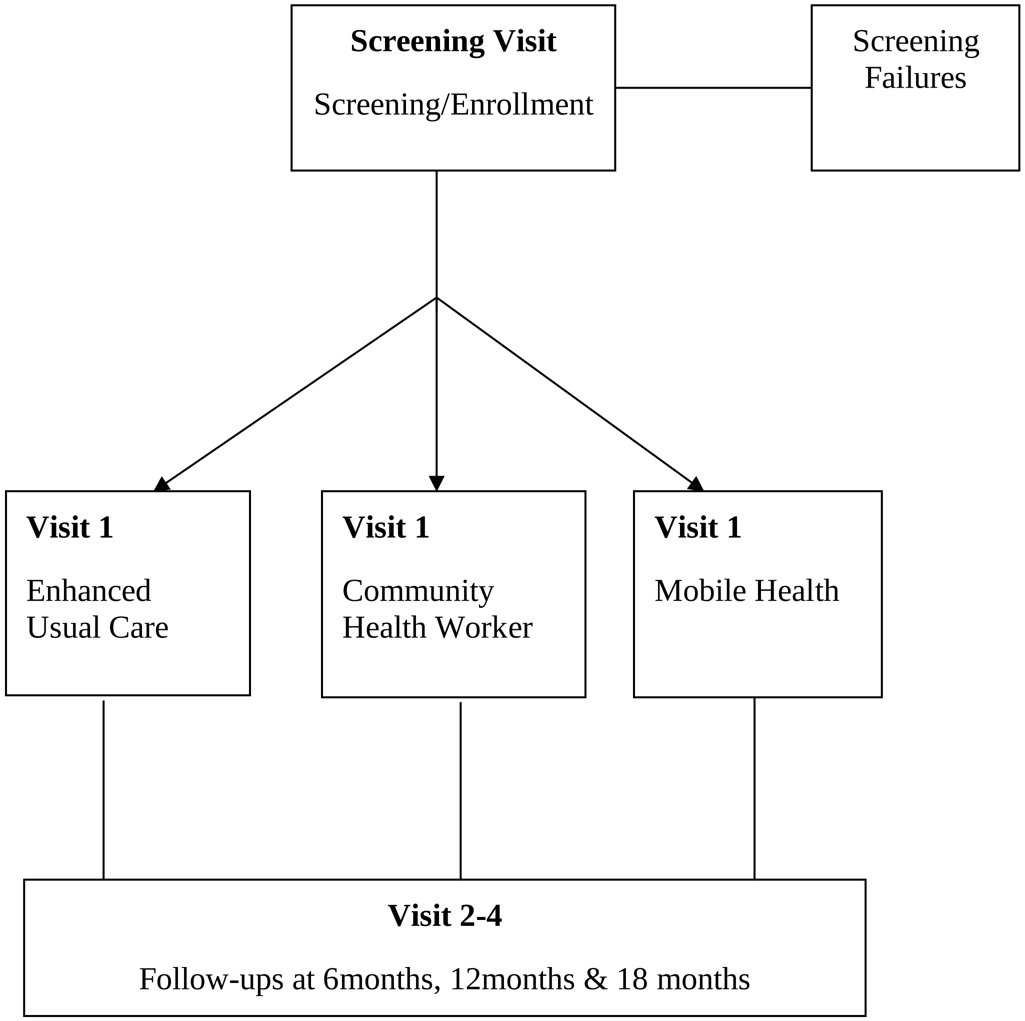

1 BACKGROUND INFORMATION AND RATIONALE

1.1 Introduction

Emerging adults with sickle cell disease (SCD) experience a seven-fold increase in mortality rates during the transition period (16-25 years of age).[1] This staggering increase in mortality and acute care utilization during this vulnerable period is partly due to difficulty coordinating care during the transition to adult care. Critical psychosocial issues further compound the vulnerability of emerging adults with SCD during the transition from pediatric to adult care[9]. Unfortunately, none of these transition intervention studies included adolescents or young adults with SCD. Patients, parents, and providers of those with SCD agree that the ability to independently perform chronic disease self-management is critical to staying healthy during this turbulent transition period.[16,17] Self-management support is a key component of the Chronic Care Model[18]. Community health worker (CHW) programs are increasingly popular and have efficacy on chronic disease self-management and system navigation. Mobile health platforms are equally popular[40], and have efficacy on self-management and adherence. Unfortunately, the effectiveness of mHealth and tailored texting among emerging adults with SCD is still unknown. Furthermore, also unknown is the comparative effectiveness of CHW programs and mHealth.

1.2 Name and Description of Investigational Product or Intervention

Control Arm: **Enhanced Usual Care**. Enhanced usual care will be standardized across sites with transition/transfer of care checklists that will be used at all sites. Enhanced usual care will minimally include (1) patient seen by the pediatric provider with the parent outside the examination room, (2) a social work consult to screen and address sociodemographic risk factors, (3) information on health insurance adequacy provided to patient, (4) adult hematologist identified, (5) adult primary care provider identified, (6) medical release signed, and (7) medical record viewable or sent to adult provider. Elements of the checklists are based on existing checklists by organizations such as the Transfer of Care Checklist developed by Got Transition/Center for Health Care Transition Improvement (www.gottransition.org), and those developed by the National Institute for Child Health Quality (http://sicklecell.nichq.org/resources/scd-transition-resources)

Intervention 1: Enhanced Usual Care + Peer Community Health Worker. The CHW program will primarily be modeled after the highly successful IMPaCT Program developed by the Penn Center for Community Health Workers [29,32,60] and CHOP's Youth CHW Program for Pediatric to Adult Transitions[61] developed by our research team, which were both developed with high levels of patient input[55,62-64]. SCD specific content and expertise from the CHW Program through the Sickle Cell Disease Association of American Philadelphia Delaware Valley Chapter and other published models[35] will be included. Components will include: 1) development of patient-centered goals and individualized action plan around self-care, symptom tracking, and transition to adult care; 2) provision of information, skills, and tips; and 3) tailored peer support using telephone calls and/or visits.

Frequency of CHW Contact: Participants will be communicating with their CHWs on a weekly basis, which is consistent with other successful community health worker protocols with published efficacy (Kangovi et al., 2016; Kangovi S, Mitra N, Grande D, & et al, 2014;

IMPaCT Community Health Worker Outpatient Care Manual, http://chw.upenn.edu/download accessed May 9, 2017).

CHW Matching: To improve feasibility of our study, the only matching criteria is that the community health worker should have personal or professional experience with chronic disease and have successfully transitioned to adult care. This aligns with previous literature that has studied the impact of peer mentors, peer navigators, and youth community health workers on chronic disease management. These studies define peer roles as unique supports provided by peers that share characteristics with the person they are supporting. These peer supports are enhanced by the formal structure of an intervention. Typically, peers are those who have personal experience with a specific behavior, health condition, or psychosocial stressor and similar characteristics as the target population (Dennis, 2003; Embuldeniya et al., 2013).

CHW Minimal Qualifications: CHWs will minimally have a high school diploma and personal experience with SCD and transitioning to adult care. **Peer CHW Training and Intervention Fidelity**. All staff members will be trained and will be required to read and adhere to a standardized study protocol manual, standardized study scripts and standardized checklists for each contact and interview with participants, and practice interviews with Co-Investigators Katherine Wu, Kyle Smith, and Samantha Luma. CHWs will not be allowed to conduct study tasks independently until they have reviewed all written and video training materials and can demonstrate complete mastery of all scripts and checklist items. The first 10 phone and in-person CHW sessions with patients will be audio-recorded, and reviewed with senior social workers, Co-Investigator Symme Trachtenberg and other research staff. In addition, a 10% random sample of all phone or in-person interviews, evenly distributed across the sequence of sessions and study sites, will be audio-recorded to ensure study fidelity. CHWs will meet weekly to discuss patient cases through videoconferencing with Co-Investigator Symme Trachtenberg, who will serve as the immediate supervisor for the CHWs. Adherence to treatment protocols will be determined by having audiotapes reviewed by trained adherence raters using an adherence checklist of key components. Adherence raters (research assistants) will assess a random sample of 25% of all sessions, evenly distributed across sequence of sessions and study arms, with re-checking for 25% of sessions, and any disagreements discussed to agreement.

End of CHW Intervention: Expectations around the time frame of the CHW and mHealth intervention will be very clear from the outset of enrollment. Consistent with previously published work with CHW (Kangovi et al., 2016; Kangovi S, Mitra N, Grande D, & et al, 2014), the expectation will be that the CHW relationship will only be 6 months, and that participants will need to identify patient goals which can be accomplished within a 6 month time period.

Intervention 2: Enhanced Usual Care + Mobile Health. All participants enrolled in the mHealth arm will download an enhanced version of iManage[113], which was developed by Co-Investigator Lori Crosby and adolescents and young adult patients with SCD, (See Screenshots). Components include: 1) development of patient-centered goals around self-care, symptom tracking, and transition to adult care; 2) provision of information, skills, and tips; 3) virtual peer support where users can encourage others to complete goals, forms teams, and interact with other youth with SCD; and 4) daily symptom tracking and visual tracking of goal completion[21]. iManage is an mHealth application that can be accessible by smartphone, tablet, or web, and has been found to be highly feasible and beneficial to emerging adults with SCD21. We will add with daily tailored texting (THRIVE2.0, Texting Health-related Resources to Inform, motiVate, and Engage)[114] based on patient-defined goals developed by Co-Investigator Lisa Schwartz, which has preliminarily found improved health promoting behaviors (such as sunscreen use) among survivors of pediatric cancer. Participants will undergo training on how to use and customize iManage during enrollment, and will have access to a helpdesk. iManage will be continuously monitored for content by research staff.

End of Intervention Period: For the mobile health intervention, we will continue to give youth access to iManage after the 6 month active intervention period, though we will not actively engage study participants to continue interacting with iManage after 6 months. While engagement with iManage may increase the heterogeneity of treatment dosage, most market mobile health interventions remain available indefinitely after purchase or download. Consequently, in keeping with the pragmatic design of this clinical trial, we will continue to give emerging adults access to iManage. Notably, most available literature on mobile health interventions shows drops in engagement with the mobile app after 3 months. Consequently, the likelihood of study participants remaining active and engaged with iManage appears relatively low. We will conduct sensitivity analysis to determine the effect of treatment dosage of iManage engagement on treatment outcomes.

1.3 Relevant Literature and Data

Background

1) ***Burden and impact of Sickle Cell Disease on the health of individuals.***

Emerging adults with sickle cell disease (SCD) experience a seven-fold increase in mortality rates during the transition period (16-25 years of age).[1] Furthermore, emerging adults with SCD also have the highest rates of hospitalizations, emergency room visits, and hospital readmissions compared to all other age groups.[2] Some of this increase is due to cumulative disease effects and increasing comorbidities due to age, such as stroke and silent infarcts.[3,4] This staggering increase in mortality and acute care utilization during this vulnerable period is partly due to difficulty coordinating care during the transition to adult

care. Patients experience changes in health insurance; systematic processes for transfer to adult care are lacking; and few comprehensive care programs for adults with SCD exist.[5,6] For example, while adherence to chronic transfusion therapy and hydroxyurea therapy can significantly decrease the risk of neurologic and other complications[7,8], poor care coordination to adult care compounded with poor patient engagement and disease self-management skills can significantly increase the risk of neurologic complications which can have devastating and long-term impacts on physical function, educational attainment, employment, income, and quality of life.

Critical psychosocial issues further compound the vulnerability of emerging adults with SCD during the transition from pediatric to adult care[9]. Transition occurs when young adults are learning to assume more responsibility for the management of their chronic disease. For example, neurologic complications can have devastating and long-term impacts on physical function, educational attainment, employment, income, and quality of life. While adherence to chronic transfusion therapy and hydroxyurea therapy can significantly decrease the risk of neurologic and other complications[7,8], access and adherence to these preventive therapies and medical follow-up deteriorate during the transition from pediatric to adult care[10]. Emerging adults with SCD are poorly prepared for transition to adult care[11], and may lack critical social support or disease-related knowledge about independently managing their SCD[9].

*2) **Gaps in Evidence for Transitions of Care between Pediatric and Adult Care Providers for Emerging Adults with SCD***

Despite the increased mortality, acute care utilization, and general vulnerability of emerging adults during the transition from pediatric to adult care, there has only been one systematic review of the efficacy of interventions for transition. This 2016 Cochrane review of transition to adult care interventions found only 4 small scientifically rigorous studies (total sample size of 238 patients), and could not draw firm conclusions about the effectiveness of the studied transition interventions on management of their chronic disease, healthcare utilization, or health outcomes because of the small sample size.[12] A 2011 systematic review found one randomized controlled trial included in this systematic review used a web- and SMS-delivered (texting) technology program intervention to improve self-management among adolescents with multiple chronic conditions and found improvements in self-efficacy and disease management[13]. Unfortunately, none of these transition intervention studies included adolescents or young adults with SCD.

Conceptual Frameworks This design is informed by both the Chronic Care Model and the SMART Model. Successful transition interventions should incorporate elements of the Social-Ecological Model of Adolescents and Young Adults Readiness to Transition (SMART model). The SMART Model, developed by Co-Investigator Schwartz, states that there are pre-existing factors and modifiable factors of patients, parents, and providers alike that contribute to transition readiness. Modifiable factors that should be targeted to help improve transition include knowledge, skills, confidence (or self-efficacy), and social supports to support chronic disease management. The SMART Model has been validated with childhood cancer survivors, parents, and providers.[14,15] Patients, parents, and providers of those with SCD agree that the ability to independently perform chronic disease self-management is critical to staying healthy during this turbulent transition period.[16,17] Self-

management behaviors refer to daily, self-motivated, collaborative activities to manage symptoms. Self-management behaviors specific to SCD include self-awareness, emotional support, nutrition, hydration, avoidance of drinking, smoking and drugs; adherence to provider orders, such as taking daily hydroxyurea or obtaining regular transfusion or chelation therapy. Self-management support is a key component of the Chronic Care Model, [18] a widely accepted framework for developing and implementing evidence-based activities to improve care for chronic illness. Consistent with the Chronic Care Model, improvements in health outcomes such as disease control, transition success, and quality of life[19] – are brought about by patients who are "activated" and engaged in self-management behaviors and partnered with a proactive healthcare team (See Figure x). In the context of individuals transitioning to adult care, more "activated" patients have higher degrees of the modifiable targets described by Schwartz' SMART Model – knowledge, self-efficacy[20] and social support[21–23] to take care of themselves. For example, self-efficacy has been linked to improved quality of life metrics among people with SCD, including decreased pain severity[24], fewer self-reported symptoms[24], and lower levels of depression, stress, and anxiety.[25]

Community health worker (CHW) programs are increasingly popular and have efficacy on chronic disease self-management and system navigation. CHW programs have efficacy on improving health outcomes in many populations (asthma, hypertension, heart disease, diabetes, and HIV), particularly in low resource areas[26–32]. One Cochrane systematic review found that CHWs improved maternal child health outcomes and management of chronic infectious diseases[33]. Another Cochrane systematic review found that CHW self-management education programs led to improvements in participants' self-efficacy, self-rated health, and cognitive symptom management.[34] In a randomized controlled trial of the IMPaCT CHW Program developed collaborator Shreya Kangovi for hospitalized patients who were low-income, uninsured, or on Medicaid, those in the CHW intervention arm were more likely to obtain timely post-hospital primary care, report high-quality discharge communication, improvements in mental health and patient activation, and were less likely to have multiple 30-day readmissions compared to controls.[29] This model has now been adapted for use in the ambulatory setting, whose effectiveness is being tested with funding provided by PCORI. CHW programs are popular with sickle cell disease centers around the country[35–38] and SCD-related funding opportunities, [39] yet their effectiveness among adolescents and young adults with SCD is unknown.

Mobile health platforms are equally popular[40], and have efficacy on self-management and adherence among both adults[41,42] and emerging adults with chronic conditions, including diabetes and liver transplant[43–46]. One Cochrane systematic review found that web-based interventions improved self-management activities among people with chronic disease, while another systematic review found that mobile text messaging interventions improved weight loss, drug adherence, and other self-management activities.[47,48] A randomized trial of patients with asthma found that a mobile app resulted in higher asthma-related quality of life scores, improved lung function, and reduced visits to the emergency department due to asthma-related complications[49]. A randomized controlled trial of MD2Me – an internet and mobile phone-delivered disease management intervention with automated SMS algorithms – found that among emerging adults with Type 1 diabetes, inflammatory bowel disease, and cystic fibrosis, those in the intervention arm had significantly improved disease self-management and health-related self-efficacy[13]. A

randomized controlled trial of MD2Me – an internet and mobile phone-delivered disease management intervention with automated SMS algorithms – found that among emerging adults with Type 1 diabetes, inflammatory bowel disease, and cystic fibrosis, those in the intervention arm had significantly improved disease self-management and health-related self-efficacy.[13] Consequently, this technology is felt to have great promise among adolescents with chronic disease given the ubiquity of smart phones.[50] Our research team has shown that emerging adults with SCD have high levels of access to technology, including high rates of smartphone ownership (e.g., 85%;[51]). Furthermore, they have strong preferences for mobile app features to improve adherence and overall SCD-related care.[51]

Unfortunately, the effectiveness of mHealth and tailored texting among emerging adults with SCD is still unknown. Furthermore, also unknown is the comparative effectiveness of CHW programs and mHealth among emerging adults with SCD during this vulnerable period, a question this clinical trial intends to answer.

1.4 Compliance Statement

This study will be conducted in full accordance all applicable Children's Hospital of Philadelphia Research Policies and Procedures and all applicable Federal and state laws and regulations including 45 CFR 46, 21 CFR Parts 50, 54, 56, 312, 314 and 812 and the Good Clinical Practice: Consolidated Guideline approved by the International Conference on Harmonisation (ICH). All episodes of noncompliance will be documented.

The investigators will perform the study in accordance with this protocol, will obtain consent and assent, and will report unanticipated problems involving risks to subjects or others in accordance with The Children's Hospital of Philadelphia IRB Policies and Procedures and all federal requirements. Collection, recording, and reporting of data will be accurate and will ensure the privacy, health, and welfare of research subjects during and after the study.

2 STUDY OBJECTIVES

The purpose of the study is to determine the comparative effectiveness of CHW programs and mHealth among emerging adults with SCD during transition versus enhanced usual care to improve health-related quality of life and acute care use for transitioning youth with SCD.

2.1 Primary Objective (or Aim)

The primary objective of this study is to compare the effectiveness of two self-management support interventions (community health workers and mobile health) versus enhanced usual care to improve health-related quality of life and acute care use for transitioning youth with SCD.

2.2 Secondary Objectives (or Aim)

The secondary objectives are to:

- Identify and quantify whether patient activation, self-management behaviors, biologic markers, and transfer to adult care are mediators of intervention treatment effects.

- Identify individual and family factors that moderate intervention treatment effects

The exploratory aim is to:

- Explore the association of enhancements to usual care on pediatric and adult acute utilization

3 INVESTIGATIONAL PLAN

3.1 General Schema of Study Design

We will conduct a pragmatic, multicenter, three-arm, open-label randomized controlled trial (RCT). An RCT is the most effective study design to minimize bias and ensure that the patients in all arms are similar in observed and unobserved characteristics. Emerging adults will be randomized 1:1:1 to a 6-month community health worker program (intervention 1), a 6-month mobile health self-management program with tailored SMS texting (intervention 2), or to usual care (control). This is non-blinded study because of the nature of the intervention prevents patients from being blinded to their study arm.

3.1.1 Screening Phase

The research team will scan hospitalized patients daily and hematology appointments weekly to identify potentially eligible patients. For non-hospitalized patients, we will confirm with the continuity hematologist that the patient can potentially transfer to adult care within the next 12 months. For hospitalized patients, we will confirm with the patient's hospital-based and ambulatory hematologist that the patient can potentially transfer to adult care within the next 12 months. Eligible patients will be flagged by the clinic staff, who will inform the patient about the study and provide more information if the patient is interested.

3.1.2 Study Treatment Phase (start of the study intervention)

The enrollment visit will include review of the details of the clinical trial and consenting of the patient.

3.1.3 Follow-ups

Patients will complete follow- up at 6 months, 12 months, and 18 months. Details of the follow up will vary based on randomization. Patients will have 6-months to complete each of the follow up surveys.

3.2 Allocation to Treatment Groups and Blinding

Random assignment will take place after enrollment and after baseline surveys. It will be planned centrally by the Children's Hospital of Philadelphia's Healthcare Analytics Unit (HAU). HAU will create a document including stratification and randomly permutated blocks of unequal sizes (to prevent providers and patients from manipulating the randomization to favor any treatment). This document will be utilized in REDCap's randomization module, which will be used for the randomization process. Certain patient characteristics – namely the treating center, age, and disease severity – may confound or influence the primary outcomes of health related quality of life and acute care utilization. To

ensure that there is an equal distribution of participants with these predictive characteristics across treatment arms, enrolled participants will be randomly assigned to a treatment arm after being stratified[58,59] by recruitment site, age (≤21 years, ≥22 years), and disease severity (mild and severe). Severe SCD disease will be determined by chart review and defined by the following: (1) Any history of stroke, (2) acute chest syndrome in the last 5 years, (3) ≥3 hospitalizations within the 3 years prior to enrollment, [52,53,54] or (4) receipt of monthly exchange transfusion therapy, defined by 2 or more exchange transfusions in an outpatient setting within 6 month period. Participants disease severity will be confirmed with the patient's hematologist.

3.3 Study Duration, Enrollment and Number of Sites

3.3.1 Duration of Study Participation

The study duration per subject will be up to18 months, with up to 6 months involving the active intervention period and 12 month follow up after.

3.3.2 Total Number of Study Sites/Total Number of Subjects Projected

The study will be conducted at multiple investigative sites in the United States.

We estimate that there are approximately 745 eligible patients across the recruitment sites over the recruitment period. It is expected that approximately 450 will be enrolled as evaluable subjects.

3.4 Study Population

3.4.1 Inclusion Criteria

1) Males or females age 17 years or older
2) Have sickle cell disease, defined as those individuals with HbSS, HbSC, HbSβ^0Thal, HbSβ^+Thal , and HbS-other genotypes
3) Receive care at a participating pediatric sickle cell disease center.
4) Appropriate for transfer to an adult hematologist within 12 months

3.4.2 Exclusion Criteria

Individuals with an intellectual disability that is severe enough that the individual would not have the capacity to interact with a mobile or web-based program even with assistance or have a conversation with a community health worker (i.e. non-verbal). Individuals without access to a mobile device, tablet or computer.

Subjects that do not meet all of the enrollment criteria may not be enrolled. Any violations of these criteria must be reported in accordance with IRB Policies and Procedures.

4 STUDY PROCEDURES

4.1 Screening Visit

Each site's investigative team will scan hospitalized patients daily and hematology appointments weekly to identify potentially eligible patients. For non-hospitalized patients, we will confirm with the continuity hematologist that the patient can potentially transfer to adult care within the next 12 months. For hospitalized patients, we will confirm with the patient's hospital-based and ambulatory hematologist that the patient can potentially transfer to adult care within the next 12 months. Eligible patients will be flagged by the clinic staff, who will inform the patient about the study and provide more information if the patient is interested. If the patient wants to enroll, enrollment visit will be conducted by study personnel. For patients enrolled during the COVID-19 pandemic electronic medical records will be reviewed to identify eligible participants. Participants eligibility will be confirmed with the patient's hematologist. Once confirmed, research staff will reach out to the patient and provide them with information about the study and complete the enrollment visit.

At the enrollment visit, we will:

- Review the clinical trial
- Obtain informed consent

4.2 Study Treatment Phase

Emerging adults will be randomized 1:1:1 to a 6-month community health worker program (intervention 1), a 6-month mobile health self-management program with tailored SMS texting (intervention 2), or to usual care (control).

4.2.1 Visit 1

At visit 1:

- Randomization of intervention will occur
- Subject will be introduced to study arm
- Baseline surveys will be taken
- Disease severity, healthcare utilization, and biologic markers will be measured from the electronic medical record

If time permits, visit 1 will occur directly after the enrollment visit. If not, it will be scheduled to occur within 2 weeks of enrollment. Participants will be compensated $50 for visit 1 through a ClinCard. During the COVID-19 pandemic, participants will be emailed the baseline survey after the screening visit. Once the baseline survey is completed participants will be contacted to inform them which intervention arm they have been assigned to.

4.2.2 Visit 2

Visit 2 will be conducted 6 months after visit 1. Participants will be offered the option to have visit 2 conducted online, by phone, or in-person. At visit 2:

- Follow up surveys will be taken. We will give participants the option to be reminded of surveys through SMS text messaging, email, or by phone. Surveys be made available through a survey link embedded within an SMS text message. We will offer participants the option to meet them at locations which are convenient for the participants (i.e. school, home, community, medical appointments).

- Disease severity, healthcare utilization, and biologic markers will be measured from the electronic medical record.

- Participants will be compensated $75 for visit 2 through a ClinCard.

4.2.3 Visit 3

Visit 3 will be conducted 12 months after visit 1. Participants will be offered the option to have visit 2 conducted online, by phone, or in-person. At visit 3:

- Follow up surveys will be taken. We will give participants the option to be reminded of surveys through SMS text messaging, email, or by phone. Surveys be made available through a survey link embedded within an SMS text message. We will offer participants the option to meet them at locations which are convenient for the participants (i.e. school, home, community, medical appointments)

- Disease severity, healthcare utilization, and biologic markers will be measured from the electronic medical record.

- Medical chart review completed by research staff

- Participants will be compensated $100 for visit 3 through a ClinCard.

4.2.4 Visit 4: End of Study

Visit will be conducted 18 months after visit 1. Participants will be offered the option to have visit 2 conducted online, by phone, or in-person. At visit 4:

- Follow up surveys will be taken. We will give participants the option to be reminded of surveys through SMS text messaging, email, or by phone. Surveys be made available through a survey link embedded within an SMS text message. We will offer participants the option to meet them at locations which are convenient for the participants (i.e. school, home, community, medical appointments).

- Disease severity, healthcare utilization, and biologic markers will be measured from the electronic medical record

- Participants will be compensated $150 for visit 4 through a ClinCard.

4.3 Subject Completion/Withdrawal**.**

Whenever a participant drops out of a research study, we will report the specific reason for dropout, in as much detail as possible; who decided that the participant would drop out; and whether the dropout involves some or all types of participation. We will attempt to continue to collect information on key outcomes on participants (electronic medical record data, billing administrative data if available) unless consent is withdrawn. All participants included in the study will be accounted for in the report, whether or not they are included in the analysis.

5 STUDY EVALUATIONS AND MEASUREMENTS

5.1 Screening and Monitoring Evaluations and Measurements

5.1.1 Medical Record Review

Include a listing of the variables that will be abstracted from the medical chart (paper or electronic).

- MRN
- first/last name
- Gender
- Date of Birth
- Zip code
- SCD genotype
- History of Intellectual Disability
- History of Depression or Anxiety
- History of Stroke or Acute Chest Syndrome
- ≥3 emergency room visits within the 3 years prior to enrollments
- Prescribed hydroxyurea therapy
- Receipt of monthly exchange transfusion therapy, defined by 2 or more exchange transfusions in an outpatient setting within 6 month period
- COVID-19 testing results
- COVID-19 antibody testing results

Biologic Marker of Disease Control (mediator) will be obtained through chart review and will include total hemoglobin level (g/dL), mean corpuscular volume (fL)[74,7], reticulocyte count (%) and fetal hemoglobin percentage[74,7]. Hemoglobin levels indicate a patient's degree of anemia. Mean corpuscular volume (fL) indicates a patient's adherence to hydroxyurea and fetal hemoglobin level. Reticulocyte count has been associated with acute stroke, with each unit percentage increase in reticulocyte count correlates with a 1.3% average increased risk in acute stroke. The level of fetal hemoglobin (HbF) expression is one of the most important modifiers of disease expression for patients with sickle cell anemia.[75] The percentage of HbF (%HbF) influences both laboratory values and clinical features of children and adults with sickle cell anemia. An elevated %HbF has been significantly associated with fewer painful

vaso-occlusive events[76], fewer episodes of acute chest syndrome[77], and reduced early mortality[76,77]

5.2 Efficacy Evaluations

5.2.1 Diagnostic Tests, Scales, Measures, etc.

Measures of Self-Management Behavior will include: (1) coping strategies using the short version of the Coping Strategies Questionnaire (Brief COPE)[67];(2) medication adherence using the Medical Adherence Measure (MAM);[65,66] an 8-item subscale about medication adherence. (3) number of no show appointments assessed through medical record review; and (4) chronic transfusion adherence will be measured by the number of scheduled simple or exchange transfusions based on patient report, medical chart review, and administrative data as described above.

Measures of Patient Activation (mediator) will include (1) level of SCD Knowledge will be assessed using the Sickle Cell Disease Knowledge Questionnaire (SCDKQ)[70]; (2) readiness to transition using the Transition Readiness Assessment Questionnaire (TRAQ)[71]; a 20-item instrument assessing skills for self-management and self-advocacy and a 4 question sub-scale from the TIP-RFT[69,70] assessing education and vocation; and (3) social support will be measured using the Medical Outcomes Study Social Support Survey (MOS-SSS)[72,73] a 19-item instrument which measures the perceived availability of social support on four subscales (emotional/informational, affectionate, tangible, and positive social interaction) using a summated rating scale.

For the **mHealth intervention**, these will include: number of days where app was accessed, number of times a day that app was accessed, number of times peer leaderboard was accessed, number of responses to SMS messages, and goal attainment progress. These measures will be tracked by downloadable usage data from all participants in the mHealth arm. For the **CHW intervention,** these will include the number of weeks with at least one contact between CHW and participant; total in-person contacts, total phone contacts, total email contacts, total SMS contacts, and goal attainment progress. These measures will be tracked by all CHW through REDCap. **Usual care process measures** will include social work consult ordered in electronic medical record and a chart review transition checklist: (1) social work consult, (2) insurance reviewed, (3) adult hematologist identified, (4) adult primary care provider identified, (5) medical release signed, (6) medical record viewable or sent to adult provider, (7) patient seen by pediatric provider alone.

Health-Related Quality of Life (primary outcome) will be assessed with the PedsQL Sickle Cell Disease Module.[52,64] and the quality of care subscale of the Adult Sickle Cell Quality of Life Measurement Information System (ASCQ-Me) measure.

The PedsQL Sickle Cell Disease Module is a well-validated for use in adolescents and young adults with chronic disease and use a Likert response scale, with higher scores indicating better HRQOL and lower SCD symptoms and problems. **The PedsQL SCD Module** is a 43-item that measures nine scales: Pain and Hurt (9 items), Pain Impact (10 items), Pain Management and Control (2 items), Worry I (5 items), Worry II (2 items),

Emotions (2 items), Treatment (7 items), Communication I (3 items), and Communication II (3 items). Average scores are calculated based on a 5-point response scale for each items and then transferred to a 0 to 100 scale with a higher score representing better quality of life. **Development:** The themes, or concepts, of the PedsQL SCD Module were initially identified through a literature review, consultation with SCD experts, individual in-depth interviews and cognitive interviews ("think aloud" to get feedback on themes) with patients with SCD 5-18 years of age and parents of children with SCD 2-18 years of age, item generation, followed by another round of cognitive interviews with patients with SCD and their parents to get feedback on how patients and parents understood each item145. **Validation:** The instrument was then validated in 243 children (aged 5-18 years) and 313 parents/caregivers of children with SCD. The internal consistency was excellent with the total scale ($\alpha = 0.95$ for patient self-report). Inter-correlations with PedsQL Generic Core Scales and PedsQL Multidimentional Fatigue Scales were medium (0.30) to large (0.50), supporting construct validity. PedsQL SCD Module Scale Scores were generally worse for patients with severe versus mild disease. Confirmatory factor analysis showed an acceptable to excellent model fit.

ASCQ-Me is a validated tool that has been widely used among adults with sickle cell disease and uses multiple choice questions to evaluate the physical, mental, and social health of the individual. We will be using the ASCQ-Me Quality of Care (ASCQ-Me QOC) subscale. This subscale is a 27-item that measures the quality of care an individual has received. **Development**: The ASCQ-Me QOC questions were developed through information from the NHLBI consumer working groups and a systematic literature review. Additionally, individual and focus group interviews were conducted with adults diagnosed with SCD as well as clinical providers. The ASCQ-Me QOC questions reflect 4 domains of health care quality-Access, Provider Communication, ED Care, and ED Pain Treatment- and are modeled after the Consumer Assessments of Healthcare Providers and Systems (CAHPS) surverys. **Validation:** The instrument was validated in 556 adults [≥18 years] with SCD. All four domains demonstrated internal consistency very well ($\alpha>0.70$ for all three). Construct validity presented (r=0.32-0.83) excellent correlations between the CAHPS global ratings and the ASCQ-Me QOC for each domain. Compared to CAHPS, Adults with SCD had worse care.

Healthcare utilization (outcomes which include acute care use and usual source of adult care) will be obtained from patient-report, the electronic medical record (EMR) of all pediatric and adult sites, and Medicaid administrative billing data. We will assess the number of emergency department visits, hospital admissions, hospital days, scheduled clinic visits, unscheduled clinic visits. We will use the Medical Adherence measure questionnaire which includes a medication adherence assessment. Acute care use will be defined as the total number of emergency department visits, hospital admission, and unscheduled clinic visits. Usual source of pediatric care will be determined by the total number of ambulatory visits in a pediatric clinic. Usual source of adult care will be determined by the total number of ambulatory visits in an adult clinic. Besides patient-reported healthcare utilization using the Medical Adherence Measure, we will also validate patient-reported data with healthcare utilization data obtained from the EMR. In particular, annualized acute care utilization, defined as the number of encounters (hospital stays and treat-and-release ED visits) over the

study period will be calculated per study participant using methodology previously described (Brousseau, Owen, Mosso, et al., JAMA 2010). Additionally, we will calculate annualized pediatric hematology ambulatory care use rate, defined as the average number of pediatric hematology ambulatory visits per year over the study period per study participant. We will also calculate annualized adult hematology ambulatory care use, defined as the average number of adult hematology ambulatory visits per year over the study period per study participant.

We will collect data related to the COVID-19 pandemic utilizing questions from the Johns Hopkins COVI-19 Community Response Survey[78] focusing on questions around COVID-19 testing, exposure, changes in violence and trauma, and safety measures. Two questions assessing reasons for foregoing care during the pandemic from the US Census Bureau Household Pulse Survey[79]. We will assess experiences with telehealth using the Telehealth Usability Questionnaire (TUQ)[80]. Each of these questions will be added to all survey time points for all participants.

STATISTICAL CONSIDERATIONS

5.3 Primary Endpoint

The primary endpoint will be the change in health-related quality of life and healthcare utilization during the duration of study participation for each subject.

5.4 Secondary Endpoints

Secondary endpoints will include the following:

- We will perform mediation analysis using structural equation modeling; this will allow us to estimate the total effect of the treatment on outcomes as a sum of direct effects and indirect effects that are mediated by patient activation, self-management behaviors, biologic markers, or transfer to adult care.
- To test the hypothesis that adults with SCD and high disease severity will moderate the effect of intervention on outcome measures at 6, 12, and 18 months, we will first evaluating whether the impact of the intervention on overall changes (six month minus baseline) differs between the high versus low severity groups.
- For the exploratory aim we will compare the demographic and clinical covariates including COVID-19 testing status of those in the enhanced usual care versus historical controls using chi-square tests (for categorical variables) and t-tests or Wilcoxon rank sum tests, as appropriate (for continuous variables). We will then compare the pediatric acute care utilization rates in the enhanced usual care versus historical control groups using the t-test or Wilcoxon rank sum test, as appropriate.

5.5 Statistical Methods

5.5.1 Baseline Data

We will first compare groups at baseline, using chi-square tests for categorical variable and traditional or nonparametric Kruskall Wallis one-way analysis of variance (ANOVA), as appropriate, for continuous variables. We will use descriptive summaries and graphical displays of the data to evaluate the distribution of and relationship between variables. When required, transformations to achieve normality (e.g. natural log) will be considered.

5.5.2 Efficacy Analysis

The initial analysis will use ANOVA to test the hypothesis of equality of changes. In other words, the three treatment groups showed no change from the end of the intervention to the baseline in the primary and secondary outcomes. If the hypothesis of equal group means is rejected (there was a change detected), 3 post-hoc tests will be applied (with Bonferroni adjustment for multiple testing) to identify pairwise differences between treatment groups.

Next, we will construct longitudinal models that include baseline, 6-month, 12-month, and 18-month measurements as outcomes. Longitudinal models account for correlation of measures within emerging adult over time; use all data collected; allow for and can be used to adjust for dropout or non-adherence to assigned treatment; can adjust for differences in

patient characteristics not balanced by randomization; can include indicator variables for clinical site; and permit continuous, count, and binary outcomes. We will build models using generalized estimating equations (GEE) or quasi-least squares (QLS)147; QLS is based on GEE, but allows for implementation of the Markov correlation structure that is appropriate if participants intermittently miss appointments (e.g. miss the 12-month visit but complete the visit at 18 months). Mixed-effects models may also be considered. For example, mixed models can be used to relax the assumption of constant variance that is required for QLS and GEE; they will therefore be useful if residual diagnostics for GEE/QLS suggest an increase in the variance of outcomes over time.

The longitudinal models will include indicator variables for visit, group, and time by visit group interaction terms. (If appropriate, time will be included as a continuous variable.) If the time by group interaction terms differ significantly from zero, this will indicate that the change over time in outcomes differs significantly between the treatment groups. In sensitivity analysis to examine the impact of treatment intensity**,** we will also evaluate the effects of the intervention stratified by intervention dosage (frequency of interaction with the community health worker or mHealth / texting program.

We will also explore comparing the treatment effects of the two intervention arms, after performing one-way ANOVA to detect any difference between groups, we will perform three tests: to compare the two interventions to each other, and to compare each intervention to control. We will use a Bonferroni correction to account for the multiple tests, so that the significance level for each test will be 0.0167 (0.05 divided by 3). The total sample of 360 evaluable subjects achieves 96% power [UP5] to detect a non-zero contrast of means of 10.00 using an F test with a 0.0167 significance level. This assumes changes of +10, 0, -10 for the three treatment groups and a common standard deviation of 19.90. This corresponds to a hypothesized effect size of 0.2051 for comparison of the two intervention arms.

To assess the effectiveness of Aim 2 the analysis will focus on the role of patient activation and self-management behaviors at 6 or 12 months as mediators of the effect of the intervention on the study outcomes. (Mediation analysis, See Figure 2.) We will perform the mediation analysis using structural equation modeling; this will allow us to estimate the total effect of the treatment on outcomes as a sum of direct effects and indirect effects that are mediated by patient activation, self-management behaviors, biologic markers, or transfer to adult care. To evaluate the role of each potential mediating variable, we will calculate the proportion of the total effect that is mediated; the ratio of the indirect effect to the direct effect; and the ratio of the total effect to the direct effect. We see also evaluate percentile and bias-corrected bootstrapped standard errors and confidence intervals for the effects[148]

To test the hypothesis that adults with SCD and high disease severity will moderate the effect of intervention on outcome measures at 6, 12, and 18 months, we will first evaluating whether the impact of the intervention on overall changes (six month minus baseline) differs between the high versus low severity groups. This will be tested by building a regression model with changes (end of intervention minus baseline) as the outcome variable, and that includes indicator variables for treatment groups, severity group (high versus low), and group by severity interaction terms (product of group and severity). If the interaction term

for a particular treatment group differs significantly from zero, this will indicate that the impact of that intervention differs according to (is moderated by) severity of disease. Next, to evaluate moderation in longitudinal models, we will fit longitudinal models with change since baseline at 6, 12, and 18 months as the outcomes; these models will also include indicator variables for treatment groups, indicator variables for severity group, and time by severity interaction terms. If the interaction terms differ significantly from zero, then this will indicate that the change since baseline depends on both treatment group and severity status (i.e. that the impact of treatment is moderated by severity of disease). We anticipate that this model will be adequate to evaluate interactions; however, it may be necessary to consider more complex models, by modifying the longitudinal models for Aim 1 to include time by severity group, severity group by intervention, and severity by time by treatment group (three way interaction) models. We will use the same analytic approach for Aim 3B**,** to test the moderating effect of age on treatment effects. Finally, to explore the moderating effects of individual and family demographic characteristics on treatment effects, we will convert all patient and family demographic variables into dichotomous variables, and approach aim 3C as we approached 3A.

For the exploratory aim we will first compare demographic and clinical covariates including COVID-19 testing status of those in the enhanced usual care versus historical controls using chi-square tests (for categorical variables) and t-tests or Wilcoxon rank sum tests, as appropriate (for continuous variables). We will then compare the pediatric acute care utilization rates in the enhanced usual care versus historical control groups using the t-test or Wilcoxon rank sum test, as appropriate. We will also use the t-test or Wilcoxon rank sum test to compare adult acute care utilization rates in the enhanced usual care versus historical controls. Covariates associated with both enhancements to usual care and utilization rates with a p-value <.05 will then be included in multivariable regression models. In addition, we will explore the following variables as potential effect modifiers: (1) Mean number of pediatric ambulatory hematology visits per year, defined as the number of pediatric ambulatory hematology visits over the 24 month study period divided by 2, calculated at the patient level. (2) Mean number of adult ambulatory hematology visits per year, defined as the number of pediatric ambulatory hematology visits over the 24 month study period divided by 2, calculated at the patient level. Effect modification will be evaluated in models for the primary outcome variables that include an indicator variable for enhanced usual care (versus usual care), the mean number of pediatric ambulatory hematology visits per year, and an enhanced usual care by mean number of pediatric ambulatory hematology visit interaction term. If the regression coefficient for the interaction term differs significantly from zero, then this will indicate that the difference in utilization rates between enhanced usual care versus usual care varies according to the mean number of pediatric hematology visits per year. The same approach will be used to evaluate the mean number of adult ambulatory hematology as an effect modifier. Depending on the distribution of the outcome measures, we will either use linear, Poisson, or negative binomial multivariate regression models to assess the association of pediatric enhancements to usual care and the outcomes, pediatric and adult acute care utilization. Poisson models would be constructed for the count of pediatric (or adult) encounters, and will include the follow-up time as an offset if the follow-up time varies between subjects. The likelihood ratio test will be used to evaluate the significance of covariates and of the indicator variable for enhanced usual care versus usual

care. In addition, the likelihood ratio test will be used to choose between the Poisson versus negative binomial models. All analyses will be performed using STATA 15 (or SAS), with two-sided tests of hypotheses and a p-value < 0.05 as the criterion for statistical significance.

5.5.3 Safety Analysis

Clinical adverse events (AEs) will be monitored throughout the study. Where issues of clinical relevance are identified by questionnaires, this information will be shared with the medical team caring for the patient.

5.6 Sample Size and Power

Aim 1: Panepinto found that among 321 children with SCD between, the standard deviation of those with severe and mild disease to be 19.9 and 17.4 on the PedsQL SCD Total Score63. Ewing found standard deviations for young adults with SCD and other hematologic conditions to be 11.32 on the PedsQL 4.0 GCS YA scale138. We assumed that there will be 360 evaluable emerging adults (from 450 enrolled), and that these will be divided 1:1:1 across three groups, yielding 120 participants in each arm. **PedsQL SCD Power Calculation (Aim 1):** This sample size will have a power of 0.97 to detect a difference of 10 points between any 2 groups in the primary outcome PedsQL SCD Module Total Score assuming a SD of 19.9 and a significance level of 0.05 for one-way ANOVA. We consider a difference of 10 points to be the smallest difference between groups that would be potentially clinically important. This was derived using the cut points for PedsQL SCD Module Pain Impact for low and high functioning patients with SCD, Panepinto found that low functioning children and a mean of 46.53 (SD 12.89), intermediate functioning children had a mean 71.76 (SD 7.79), while the high functioning children had mean scores of 89 (SD 9.4).[53] **PedsQL 4.0 GCS Power Calculation (Aim 1):** This sample size will also have a power of .99 to detect a 5 point change in PedsQL 4.0 GCS YA scale assuming a SD 11.32 and a significance level of 0.05 for a one-way ANOVA. We consider a difference of 5 points to be the smallest difference between groups that would be potentially clinically important.[78,79]

Aim 2 Power Calculation: Here we present sample size calculations obtained using methods from Fritz and MacKinnon (2007). We will perform mediation analysis to estimate the total effect of the treatment on outcomes as a sum of total and indirect effects that are mediated by patient activation, self-management behaviors, biologic markers, or transfer to adult care. To evaluate the role of each potential mediating variable, we will calculate the proportion of the total effect that is mediated; the ratio of the indirect effect to the direct effect; and the ratio of the total effect to the direct effect. We see also evaluate percentile and bias-corrected bootstrapped standard errors and confidence intervals for the effects. The percentile bootstrap test of mediation will take a random sample from the original data with replacement. The percentile bootstrap test computes 95% confidence intervals based on the 0.025 and 0.975 percentiles of the bootstrap sample distribution. Significant mediation is said to occur at the 0.05 level of significance, if the 95% percentile bootstrap confidence interval does not contain zero. The bias corrected bootstrap corrects the percentile confidence intervals so that the confidence interval is symmetric. As shown in Table 3 of Fritz and MacKinnon (2007), sample sizes of 406 and 404 respectively, are required to

detect small alpha (impact of intervention on mediator) and medium beta paths (mediator on outcome, adjusted for intervention) with 80% power. Sample sizes of 404 (for percentile) and 391 (for bias corrected) are required to detect medium alpha and small beta paths with 80% power. The required sample sizes drop to 78 (for percentile) and 71 (for bias corrected) to detect medium alpha and medium beta effects with 80% power. With a sample size of 402 we will therefore be well powered to detect at least medium alpha and beta paths with 80% power.

PedsQL SCD Power Calculation for Severe SCD Disease Subgroup (Aim 3): We assume that approximately 65% of the study population size will be considered to have severe disease based on CHOP registry estimates of those taking hydroxyurea or on chronic transfusion, yielding approximately 78 participants per treatment arm. This sample size will have a power of 0.88 to detect a difference of 10 points between any 2 groups in the primary outcome PedsQL SCD Module Total Score assuming a SD of 19.9 and a significance level of 0.05 for one-way ANOVA. **PedsQL 4.0 GCS Power Calculation for Severe SCD Disease Subgroup (Aim 3):** This sample size will also have a power of 0.79 to detect a 5 point change in PedsQL 4.0 GCS YA scale assuming a SD 11.32 and a significance level of 0.05 for a one-way ANOVA.

Aim 4 Sample Size: We do not have preliminary data on which to power this exploratory aim.

6 SAFETY MANAGEMENT

6.1 Clinical Adverse Events

Clinical adverse events (AEs) will be monitored throughout the study.

6.2 Adverse Event Reporting

Since the study procedures are not greater than minimal risk, SAEs are not expected. If any unanticipated problems related to the research involving risks to subjects or others happen during the course of this study (including SAEs) they will be reported to the IRB in accordance with CHOP IRB SOP 408: Unanticipated Problems Involving Risks to Subjects. AEs that are not serious but that are notable and could involve risks to subjects will be summarized in narrative or other format and submitted to the IRB at the time of continuing review.

7 STUDY ADMINISTRATION

7.1 Treatment Assignment Methods

7.1.1 Randomization

Random assignment will take place after enrollment and after baseline surveys. It will be planned centrally by the Children's Hospital of Philadelphia's Healthcare Analytics Unit (HAU). HAU will create a spreadsheet of randomly permutated blocks of unequal sizes (to prevent providers and patients from manipulating the randomization to favor any treatment). Certain patient characteristics – namely the treating center, age, and disease severity – may confound or influence the primary outcomes of health-related quality of life and acute care utilization. To ensure that there is an equal distribution of participants with these predictive characteristics across treatment arms, enrolled participants will be randomly assigned to a treatment arm after being stratified[58,59] by recruitment site , age (≤21 years, ≥22 years), and disease severity (mild and severe), resulting in 12 different randomization strata. Severe SCD disease will be determined by chart review and defined by the following: (1) Any history of stroke, (2) acute chest syndrome in the last 5 years, (3) ≥3 hospitalizations within the 3 years prior to enrollment, [52,53,54] or (4) receipt of monthly exchange transfusion therapy, defined by 2 or more exchange transfusions in an outpatient setting within 6 month period. The spreadsheet will be used to create a randomization module within REDCap, which will be closely monitored by HAU.

7.1.2 Blinding

This is non-blinded study because of the nature of the intervention prevents patients from being blinded to their study arm.

7.2 Data Collection and Management

All survey data will be entered and stored in REDCap. All data linkage will be conducted at CHOP via staff at the Healthcare Analytics Unit. All participating sites will send data via a secure FTP website. These data will be stored on the secure Virtual Desktop environment, where all linkage and data manipulation will take place. Linkage will be conducted using

MRN, first/last name , date of birth, and gender, where appropriate. To ensure confidentiality of patient information, after a master file of linked data is created, this dataset will be de-identified. Each participant will be assigned a unique study identification number. Once assigned, all data will be stripped of identifiers and files will be catalogued using the assigned unique patient identifier only. Only trained study staff members will have access to this information. A separate linking file will be kept in a password protected file on a password protected computer.

7.3 Confidentiality

The study team will make every effort to maintain patient confidentiality and has strict data security measures in place to ensure that patient information remain confidential. Study team standards are in accordance with institutional policies and HIPAA on subject privacy. When data is shared with the investigative team during implementation and dissemination of results, data will be presented in the aggregate. Any information shared on individual participants will be shared using unique study identification number only.

The study is of minimal risk because the interventions are designed to increase the effectiveness of transition from pediatric to adult health care settings for young adults with SCD disease. Participants will be given access to study team members as a means to voice any questions, concerns, or reservations throughout the study. Contact information will be provided at the time of consent should any specific questions arise, the study team will intervene to address problems including, if needed, a change or discontinuation of study procedures. The CHOP IRB will be promptly informed of any concerns.

7.4 Regulatory and Ethical Considerations

7.4.1 Data and Safety Monitoring Plan

The Principal Investigator will be responsible for monitoring the safety of study participants and complying with all reporting requirements. Any adverse events will be reported immediately to the CHOP IRB.

7.4.2 Risk Assessment

The proposed project involves in the collection of information from young adult patients. The risk of participation is considered minimal. Potential risks include: breach of confidentiality of personal health information and the risk of patients feeling uncomfortable completing study measures. The study team has taken steps to mitigate these risks. The breach of confidentiality has been made a minimal risk by assigning participants a unique study identification number, de-identifying the data set, storing data on secure, password protected, computers, and completing all data analyses and one study site. The study team has mitigated the risk of patients feeling uncomfortable with study measures by including language in patient consent stating that all participation is voluntary; patients will be allowed to stop participating in the study at any time. Should any further concerns arise throughout the study; the study team will immediately notify the appropriate parties, including the CHOP IRB, who will serve as the IRB of record. We expect adverse consequences to be rare and unlikely.

An additional risk is that patients randomized to the mHealth application arm may use the application, which is intended for non-urgent communication with peers) for urgent issues to be handled by a health care provider. This risk will be mitigated through direct instruction to patients at the time of consent and randomization to the mHealth application group, constant monitoring of peer-to-peer communication on the mHealth application by study staff, and a clear internal plan on how to facilitate appropriate communication within the application. Additionally, there will be a direct link to contact appropriate study staff within the mHealth application. Study staff who receive communication from participants will route the information to the study site social worker or medical director, where appropriate.

Participants randomized to the CHW arm of the trial are at risk for communicating about urgent health issues to the CHW, a study team member not trained in medicine. This risk will be mitigated through direct instruction to patients at the time of consent and randomization to the CHW group and reinforcement and reminders from the CHW that urgent medical questions should be addressed by a medical provider. A selection of CHW intervention meetings will be audio recorded to ensure fidelity across sites. These records will not contain patient identifiers and will be transferred to CHOP from the other study sites via secure FTP website. When appropriate, CHWs may refer patients who have medical questions to their medical team or study site social worker.

The study team will make clear to patients that participation in all aspects of the proposed study is completely voluntary. The study team will make every effort to maintain patient confidentiality and has strict data security measures in place to ensure that patient information remain confidential. Study team standards are in accordance with institutional policies and HIPAA on subject privacy. When data is shared with the investigative team during implementation and dissemination of results, data will be presented in the aggregate. Any information shared on individual participants will be shared using unique study identification number only.

7.4.4 Potential Benefits of Trial Participation

Patients may benefits directly from inclusion in this study if they utilize the interventions to their full potential. However, this cannot be guaranteed. The results of this study may assist hematology health care providers and SCD patients in the transition from pediatric to adult health care systems. This model may be replicated in different care settings and other diseases and thus generate generalizable knowledge. Further, the information gathered from this study will be disseminated widely, including to peer-reviewed journals, lay publications, and presentations at scientific conferences.

7.4.5 Risk-Benefit Assessment

The potential risks associated with study procedures are minimal. Results gained from this comparative effectiveness study will build the foundation for transition in patients with SCD and inform the development of transition models for other diseases.

7.5 Recruitment Strategy

A robust recruitment plan is in place in order to ensure maximum participation. Each site’s investigative team will scan hospitalized patients daily and hematology appointments weekly to identify potentially eligible patients. For non-hospitalized patients, we will confirm with the continuity hematologist that the patient can potentially transfer to adult care within the next 12 months. For hospitalized patients, we will confirm with the patient’s hospital-based and ambulatory hematologist that the patient can potentially transfer to adult care within the next 12 months. There will be multiple options for enrollment to meet the needs of the potentially participants. Patients who are not hospitalized will be called before the clinic visit and inform eligible patients about the study. We will offer the following options for enrollment:

a) During the upcoming hematologist appointment;
b) Within a week after the upcoming hematologist appointment;
c) During any upcoming non-hematologist appointment;
d) Via phone.

In addition, we will utilize CHOP Recruitment Enhancement Core (REC), which provides assistance with recruitment plan development and may assist in identifying and contacting potential participants using the CRU, the CHOP Recruitment Registry, social media and internal communication resources. The REC also engages community partners and facilitates outreach on behalf of the research Institute and CHOP research studies. With support of the REC, we will utilize various recruitment strategies including emails to families with language to opt-out from future contact, internal communications (i.e. This Week @CHOP, tear pads that will be displayed in Main Hospital at CHOP), a recruitment video, and social media. Study information displayed on the resources or in the email communication will be consistent with the approved IRB recruitment flyer, with minor modifications to simplify/condense language based on the type of resource or communication. Research Communications (RC) manages and maintains the official social media accounts for the Children's Hospital of Philadelphia Research Institute. The department will review all suggested subject recruitment messages, posts and images submitted through REC and approved by the IRB, determining whether the posts are consistent with the overarching mission and purpose of the social media channels and are appropriate for the respective audiences. RC will also slightly modify submitted content as needed to maintain consistent voice, tone, and approach across social media channels, determine the publishing schedule for posts, and monitor feedback for posts, and relay any comments/questions to investigators and/or study teams about a particular study. Individuals made aware of the study through REC resources will be provided with information to reach out to the investigators for further information about study participation. As a result, the study team will not have access to PHI for these individuals unless and until they contact the study team directly.

In efforts to reach all eligible participants, those identified as eligible will also receive a recruitment letter and recruitment flyer via mail describing the details of the study and providing study staff contact information.

7.6 Informed Consent/Assent and HIPAA Authorization

There will be multiple options for enrollment to meet the needs of the potentially participants. Patients who are not hospitalized will be called before the clinic visit and inform eligible patients about the study. We will offer the following options for enrollment:
a) During the upcoming hematologist appointment;
b) Within a week after the upcoming hematologist appointment;
c) During any upcoming non-hematologist appointment;
d) Via phone.

At the ambulatory hematology visit, we will verify eligibility and prior consent (when obtained by phone) prior to participant enrollment. Randomization to treatment arm

stratified by study site, age, and disease severity will be utilized. A research study team member will introduce the patient to the intervention or control.

Consent will be obtained by study coordinator or enroller trained according to HSPP protocol. Consent will be obtained in a private medical office room with ample time to discuss and assess understanding prior to obtaining consent. Participants will be asked to review and read the consent with the research personnel prior to signing. All standard procedures will be followed to maintain informed consent. Consent will also be obtained via e-consent using REDCap. For e-consent, the study will be described via phone and consent form reviewed with the participant. Once finished, the participant will provide an e-signature via REDCap. Participants will be emailed a copy of the e-consent form when possible, if not they will be mailed a copy.

Subjects who are 17-years old at the time of enrollment will be re-consented at the age of 18 in person at an upcoming appointment, via phone or via REDCap using an e-consent if they have not been selected for audio-recording of CHW interactions. Those subjects who have been selected for audio recording will be re-consented by the CHW. Subjects enrolled at Cincinnati Children's Hospital Medical Center that need to be re-consented will only provide re-consent in person (written). Subjects enrolled at Connecticut Children's Medical Center that need to be re-consented will only provide re-consent in person (written) or via REDCap using e-consent. All subjects will be given a copy of the consent. The original will be kept in a secure office.

7.6.1 Waiver of Parental Permission

A waiver of parental permission is requested for those subjects who are 17-years old, as this study focuses on transition in care to a non-pediatric healthcare provider. This research involves no more than minimal risk to participants and this waiver will not adversely affect the rights of the participants.

7.6.2 Waiver of documentation of consent and HIPAA Authorization

A waiver of documentation of consent is requested for those subjects who are 17-years old at the time of enrollment and turn 18 during the study period. Re-consent will be obtained either via phone, an e-consent via REDCap, or at an upcoming appointment after their 18th birthday and prior to the next study visit. A waiver of documentation of consent s also requested for those unable to make hematology appointments due to concerns related to the risk of coronavirus. Those subjects who have been selected for audio-recording of CHW interactions will be re-consented in person.

7.7 Payment to Subjects/Families

7.7.1 Payments to subject for time, effort and inconvenience (i.e. compensation)

Participants will receive incentives for completing surveys throughout the study period: $50 (baseline), $75 (6 month), $100 (12 month), $150 (18 month). Participant must complete all surveys to receive compensation at each of the time points.

Participants randomized to the mobile health application (Group #2) can receive up to $3 a month for engaging with the app during the study. For 3 consecutive days participants can

earn 0.30 cents. For longer streaks of engagement, the incentive increases as follows 6 days = 0.60 cents, 12 days = $1.20, 18 days = $1.80, and 30 days = $3. After 3 months of consecutive adherence, participants can earn an additional $5, after 6 months of consecutive adherence participants can earn an additional $11.

Participant incentives will be managed through ClinCards, which are more convenient to use than traditional cash, check, gift cards. A ClinCard is a web based, reloadable, debit card that automates reimbursements for participants who are engaged in clinical research. ClinCards minimize the risk of theft and participants can receive payments faster and through direct deposits if desired. Fair compensation was determined by our patient and parent Co-Investigators.

8 PUBLICATION

The information gathered from this study will be disseminated widely, including to peer-reviewed journals, lay publications, and presentations at scientific conferences.

9 REFERENCES

1. Paulukonis ST, Eckman JR, Snyder AB, et al. Defining Sickle Cell Disease Mortality Using a Population-Based Surveillance System, 2004 through 2008. *Public Health Rep Wash DC 1974*. 2016;131(2):367-375.

2. Brousseau DC, Owens PL, Mosso AL, Panepinto JA, Steiner CA. Acute care utilization and rehospitalizations for sickle cell disease. *JAMA*. 2010;303(13):1288-1294. doi:10.1001/jama.2010.378

3. Ohene-Frempong K, Weiner SJ, Sleeper LA, et al. Cerebrovascular accidents in sickle cell disease: rates and risk factors. *Blood*. 1998;91(1):288-294.

4. DeBaun MR, Armstrong FD, McKinstry RC, Ware RE, Vichinsky E, Kirkham FJ. Silent cerebral infarcts: a review on a prevalent and progressive cause of neurologic injury in sickle cell anemia. *Blood*. 2012;119(20):4587-4596. doi:10.1182/blood-2011-02-272682

5. DeBaun MR, Telfair J. Transition and sickle cell disease. *Pediatrics*. 2012;130(5):926-935. doi:10.1542/peds.2011-3049

6. Jordan L, Swerdlow P, Coates T. Systematic Review of Transition From Adolescent to Adult Care in Patients With Sickle Cell Disease. *J Pediatr Hematol Oncol*. 2013;35(3):165-169.

7. Jones AP, Davies SC, Olujohungbe A. Hydroxyurea for sickle cell disease. In: *The Cochrane Library*. John Wiley & Sons, Ltd; 2001. http://proxy.library.upenn.edu:2154/doi/10.1002/14651858.CD002202/full. Accessed December 10, 2016.

8. Wang WC, Dwan K. Blood transfusion for preventing primary and secondary stroke in people with sickle cell disease. In: *The Cochrane Library*. John Wiley & Sons, Ltd; 2013. http://proxy.library.upenn.edu:2154/doi/10.1002/14651858.CD003146.pub2/full. Accessed December 10, 2016.

9. Crosby LE, Quinn CT, Kalinyak KA. A Biopsychosocial Model for the Management of Patients With Sickle-Cell Disease Transitioning to Adult Medical Care. *Adv Ther*. 2015;32(4):293-305. doi:10.1007/s12325-015-0197-1

10. Blinder MA, Vekeman F, Sasane M, Trahey A, Paley C, Duh MS. Age-related treatment patterns in sickle cell disease patients and the associated sickle cell complications and healthcare costs. *Pediatr Blood Cancer*. 2013;60(5):828-835. doi:10.1002/pbc.24459

11. Wojciechowski EA, Hurtig A, Dorn L. A natural history study of adolescents and young adults with sickle cell disease as they transfer to adult care: a need for case management services. *J Pediatr Nurs*. 2002;17(1):18-27.

12. Campbell F, Biggs K, Aldiss SK, et al. Transition of care for adolescents from paediatric services to adult health services. *Cochrane Database Syst Rev*. 2016;2016(4). http://dx.doi.org/10.1002/14651858.CD009794.pub2. Accessed December 15, 2016.

13. Huang JS, Terrones L, Tompane T, et al. Preparing Adolescents With Chronic Disease for Transition to Adult Care: A Technology Program. *PEDIATRICS*. 2014;133(6):e1639-e1646. doi:10.1542/peds.2013-2830

14. Schwartz LA, Tuchman LK, Hobbie WL, Ginsberg JP. A social-ecological model of readiness for transition to adult-oriented care for adolescents and young adults with chronic health conditions. *Child Care Health Dev*. 2011;37(6):883-895. doi:10.1111/j.1365-2214.2011.01282.x

15. Schwartz LA, Brumley LD, Tuchman LK, et al. Stakeholder Validation of a Model of Readiness for Transition to Adult Care. *JAMA Pediatr*. 2013;167(10):939. doi:10.1001/jamapediatrics.2013.2223

16. Andemariam B, Owarish-Gross J, Grady J, Boruchov D, Thrall RS, Hagstrom JN. Identification of risk factors for an unsuccessful transition from pediatric to adult sickle cell disease care. *Pediatr Blood Cancer*. 2014;61(4):697-701. doi:10.1002/pbc.24870

17. Sobota A, Neufeld EJ, Sprinz P, Heeney MM. Transition from pediatric to adult care for sickle cell disease: Results of a survey of pediatric providers. *Am J Hematol*. 2011;86(6):512-515. doi:10.1002/ajh.22016

18. Bodenheimer T, Wagner EH, Grumbach K. Improving Primary Care for Patients With Chronic Illness: The Chronic Care Model, Part 2. *JAMA*. 2002;288(15):1909-1914. doi:10.1001/jama.288.15.1909

19. Matthie N, Jenerette C, McMillan S. Role of Self-Care in Sickle Cell Disease. *Pain Manag Nurs*. 2015;16(3):257-266. doi:10.1016/j.pmn.2014.07.003

20. Barlow J, Wright C, Sheasby J, Turner A, Hainsworth J. Self-management approaches for people with chronic conditions: a review. *Patient Educ Couns*. 2002;48(2):177-187. doi:10.1016/S0738-3991(02)00032-0

21. Crosby LE, Ware RE, Goldstein A, et al. Development and evaluation of iManage: A self-management app co-designed by adolescents with sickle cell disease. *Pediatr Blood Cancer*. August 2016:n/a-n/a. doi:10.1002/pbc.26177

22. Sin M-K, Kang D-H, Weaver M. Relationships of asthma knowledge, self-management, and social support in African American adolescents with asthma. *Int J Nurs Stud*. 2005;42(3):307-313. doi:10.1016/j.ijnurstu.2004.06.013

Chlebowy DO, Garvin BJ. Social support, self-efficacy, and outcome expectations: impact on self-care behaviors and glycemic control in Caucasian and African American adults with type 2 diabetes. *Diabetes Educ*. 2006;32(5):777-786. doi:10.1177/0145721706291760

24. Clay OJ, Telfair J. Evaluation of a disease-specific self-efficacy instrument in adolescents with sickle cell disease and its relationship to adjustment. *Child Neuropsychol J Norm Abnorm Dev Child Adolesc*. 2007;13(2):188-203. doi:10.1080/09297040600770746

25. Edwards R, Telfair J, Cecil H, Lenoci J. Self-efficacy as a predictor of adult adjustment to sickle cell disease: one-year outcomes. *Psychosom Med*. 2001;63(5):850-858.

26. Krieger J, Takaro TK, Song L, Beaudet N, Edwards K. A Randomized Controlled Trial of Asthma Self-management Support Comparing Clinic-Based Nurses and In-Home Community Health Workers: The Seattle–King County Healthy Homes II Project. *Arch Pediatr Adolesc Med*. 2009;163(2):141-149. doi:10.1001/archpediatrics.2008.532

27. Palmas W, March D, Darakjy S, et al. Community Health Worker Interventions to Improve Glycemic Control in People with Diabetes: A Systematic Review and Meta-Analysis. *J Gen Intern Med*. 2015;30(7):1004-1012. doi:10.1007/s11606-015-3247-0

28. Rosenthal EL, Brownstein JN, Rush CH, et al. Community Health Workers: Part Of The Solution. *Health Aff (Millwood)*. 2010;29(7):1338-1342. doi:10.1377/hlthaff.2010.0081

29. Kangovi S, Mitra N, Grande D, et al. Patient-centered community health worker intervention to improve posthospital outcomes: A randomized clinical trial. *JAMA Intern Med*. 2014;174(4):535-543. doi:10.1001/jamainternmed.2013.14327

30. Gary TL, Bone LR, Hill MN, et al. Randomized controlled trial of the effects of nurse case manager and community health worker interventions on risk factors for diabetes-related complications in urban African Americans. *Prev Med*. 2003;37(1):23-32. doi:10.1016/S0091-7435(03)00040-9

31. Gibbons MC, Tyus NC. Systematic Review of U.S.-Based Randomized Controlled Trials Using Community Health Workers. *Prog Community Health Partnersh Res Educ Action*. 2007;1(4):371-381. doi:10.1353/cpr.2007.0035

32. Kangovi S, Carter T, Charles D, et al. Toward A Scalable, Patient-Centered Community Health Worker Model: Adapting the IMPaCT Intervention for Use in the Outpatient Setting. *Popul Health Manag*. March 2016. doi:10.1089/pop.2015.0157

33. Lewin S, Munabi-Babigumira S, Glenton C, et al. Lay health workers in primary and community health care for maternal and child health and the management of infectious diseases. In: *The Cochrane Library*. John Wiley & Sons, Ltd; 2010. http://proxy.library.upenn.edu:2154/doi/10.1002/14651858.CD004015.pub3/full. Accessed December 16, 2016.

Foster G, Taylor SJ, Eldridge S, Ramsay J, Griffiths CJ. Self-management education programmes by lay leaders for people with chronic conditions. In: *The Cochrane Library*. John Wiley & Sons, Ltd; 2007. http://proxy.library.upenn.edu:2154/doi/10.1002/14651858.CD005108.pub2/full. Accessed December 6, 2016.

35. Hsu LL, Green NS, Donnell Ivy E, et al. Community Health Workers as Support for Sickle Cell Care. *Am J Prev Med*. 2016;51(1, Supplement 1):S87-S98. doi:10.1016/j.amepre.2016.01.016

36. Community Health Workers Provide a Safety Net for Patients with Sickle Cell Disease | The Improvement Quotient. http://www.nichq.org/blog/2016/september/community_health_workers_sickle_cell_disease. Accessed December 15, 2016.

37. Fisher KA. Community Health Workers. http://www.hopkinsmedicine.org/medicine/sickle/chw/. Accessed December 15, 2016.

38. Sickle Cell Foundation of Georgia, Inc. Meet Your New Sickle Cell Disease Community Health Workers - Sickle Cell Foundation of Georgia, Inc. http://sicklecellga.org/meet-your-new-sickle-cell-disease-community-health-workers/. Accessed December 17, 2016.

39. Sickle Cell Disease Newborn Screening Follow-Up Program. https://mchb.hrsa.gov/fundingopportunities/?id=f3f0739a-466d-434a-ab53-4f266b114cb6. Accessed December 16, 2016.

40. Research SM. mHealth Market Expected to Reach 109.16 Billion by 2022 - Scalar Market Research. http://www.prnewswire.com/news-releases/mhealth-market-expected-to-reach-10916-billion-by-2022---scalar-market-research-603764326.html. Accessed December 17, 2016.

41. Free C, Phillips G, Galli L, et al. The Effectiveness of Mobile-Health Technology-Based Health Behaviour Change or Disease Management Interventions for Health Care Consumers: A Systematic Review. *PLOS Med*. 2013;10(1):e1001362. doi:10.1371/journal.pmed.1001362

42. Holtz B, Lauckner C. Diabetes Management via Mobile Phones: A Systematic Review. *Telemed E-Health*. 2012;18(3):175-184. doi:10.1089/tmj.2011.0119

43. Militello LK, Kelly SA, Melnyk BM. Systematic Review of Text-Messaging Interventions to Promote Healthy Behaviors in Pediatric and Adolescent Populations: Implications for Clinical Practice and Research. *Worldviews Evid Based Nurs*. 2012;9(2):66-77. doi:10.1111/j.1741-6787.2011.00239.x

44. Franklin VL, Waller A, Pagliari C, Greene SA. A randomized controlled trial of Sweet Talk, a text-messaging system to support young people with diabetes. *Diabet Med J Br Diabet Assoc*. 2006;23(12):1332-1338. doi:10.1111/j.1464-5491.2006.01989.x

45. Miloh T, Annunziato R, Arnon R, et al. Improved adherence and outcomes for pediatric liver transplant recipients by using text messaging. *Pediatrics*. 2009;124(5):e844-850. doi:10.1542/peds.2009-0415

46. Huang JS, Terrones L, Tompane T, et al. Preparing Adolescents With Chronic Disease for Transition to Adult Care: A Technology Program. *Pediatrics*. 2014;133(6):e1639-e1646. doi:10.1542/peds.2013-2830

47. Pal K, Eastwood SV, Michie S, et al. Computer-based diabetes self-management interventions for adults with type 2 diabetes mellitus. In: *The Cochrane Library*. John Wiley & Sons, Ltd; 2013. http://proxy.library.upenn.edu:2154/doi/10.1002/14651858.CD008776.pub2/full. Accessed December 16, 2016.

48. de Jongh T, Gurol-Urganci I, Vodopivec-Jamsek V, Car J, Atun R. Mobile phone messaging for facilitating self-management of long-term illnesses. In: *The Cochrane Library*. John Wiley & Sons, Ltd; 2012. http://proxy.library.upenn.edu:2154/doi/10.1002/14651858.CD007459.pub2/full. Accessed December 16, 2016.

49. Liu W-T, Huang C-D, Wang C-H, Lee K-Y, Lin S-M, Kuo H-P. A mobile telephone-based interactive self-care system improves asthma control. *Eur Respir J*. 2011;37(2):310-317. doi:10.1183/09031936.00000810

50. Amanda Lenhart. Teens, Social Media & Technology Overview 2015. *Pew Res Cent Internet Sci Tech*. April 2015. http://www.pewinternet.org/2015/04/09/teens-social-media-technology-2015/. Accessed December 15, 2016.

51. Badawy SM, Thompson AA, Liem RI. Technology Access and Smartphone App Preferences for Medication Adherence in Adolescents and Young Adults With Sickle Cell Disease. *Pediatr Blood Cancer*. 2016;63(5):848-852. doi:10.1002/pbc.25905

52. Panepinto JA, Torres S, Bendo CB, et al. PedsQLTM Sickle Cell Disease Module: Feasibility, Reliability and Validity. *Pediatr Blood Cancer*. 2013;60(8):1338-1344. doi:10.1002/pbc.24491

53. Beverung LM, Varni JW, Panepinto JA. Clinically meaningful interpretation of pediatric health-related quality of life in sickle cell disease. *J Pediatr Hematol Oncol*. 2015;37(2):128-133. doi:10.1097/MPH.0000000000000177

54. *Evidence-Based Management of Sickle Cell Disease: Expert Panel Report*. U.S. Department of Health and Human Services; National Institutes of Health; National Heart, Lung, and Blood Institute.; 2014.

55. Kangovi S, Barg FK, Carter T, Long JA, Shannon R, Grande D. Understanding Why Patients Of Low Socioeconomic Status Prefer Hospitals Over Ambulatory Care. *Health Aff (Millwood)*. 2013;32(7):1196-1203. doi:10.1377/hlthaff.2012.0825

56. Torgerson DJ. Contamination in trials: is cluster randomisation the answer? *BMJ*. 2001;322(7282):355-357.

57. Hahn S, Puffer S, Torgerson DJ, Watson J. Methodological bias in cluster randomised trials. *BMC Med Res Methodol*. 2005;5:10. doi:10.1186/1471-2288-5-10

58. Zelen M. The randomization and stratification of patients to clinical trials. *J Chronic Dis*. 1974;27(7):365-375. doi:10.1016/0021-9681(74)90015-0

59. Kernan WN, Viscoli CM, Makuch RW, Brass LM, Horwitz RI. Stratified Randomization for Clinical Trials. *J Clin Epidemiol*. 1999;52(1):19-26. doi:10.1016/S0895-4356(98)00138-3

60. Steinway C, Szalda DE, Trachtenberg S, Greenberg A, Wu, Katherine, Jan, Sophia. Multidisciplinary Intervention Navigation Team (MINT): A Clinical Service for Pediatric to Adult Medical Systems Transitions for Medically Complex Young Adults. In: *Oral Presentation*. Denver, Colorado; 2016.

61. Brown EJ, Kangovi S, Sha C, et al. Exploring the Patient and Staff Experience With the Process of Primary Care. *Ann Fam Med*. 2015;13(4):347-353. doi:10.1370/afm.1808

62. Kangovi S, Grande D, Carter T, et al. The use of participatory action research to design a patient-centered community health worker care transitions intervention. *Healthcare*. 2014;2(2):136-144. doi:10.1016/j.hjdsi.2014.02.001

63. Greenberg AS, Szalda D, Trachtenberg S, et al. Transfer Engagement Lessons Learned (TELL): Using Patient Perspectives to Inform and ImproveTransition Processes. *J Adolesc Health*. 2016;58(2):S75.

64. Crosby LE, Ware RE, Goldstein A, et al. Development and evaluation of iManage: A self-management app co-designed by adolescents with sickle cell disease. *Pediatr Blood Amp Cancer*. 2017;64(1):139-145. doi:10.1002/pbc.26177

65. Zelikovsky N, Schast AP. Eliciting accurate reports of adherence in a clinical interview: development of the Medical Adherence Measure. *Pediatr Nurs*. 2008;34(2):141-146.

66. Zelikovsky N, Schast AP, Palmer J, Meyers KEC. Perceived barriers to adherence among adolescent renal transplant candidates. *Pediatr Transplant*. 2008;12(3):300-308. doi:10.1111/j.1399-3046.2007.00886.x

67. Gil KM, Williams DA, Thompson RJ, Kinney TR. Sickle Cell Disease in Children and Adolescents: The Relation of Child and Parent Pain Coping Strategies to Adjustment. *J Pediatr Psychol*. 1991;16(5):643-663. doi:10.1093/jpepsy/16.5.643

68. Sobota A, Akinlonu A, Champigny M, et al. Self-Reported Transition Readiness among Young Adults with Sickle Cell Disease. *J Pediatr Hematol Oncol*. 2014;36(5):389-394. doi:10.1097/MPH.0000000000000110

69. Treadwell M, Johnson S, Sisler I, et al. Self-efficacy and readiness for transition from pediatric to adult care in sickle cell disease. *Int J Adolesc Med Health*. 2016;28(4). doi:10.1515/ijamh-2015-0014

70. Treadwell M, Johnson S, Sisler I, et al. Development of a sickle cell disease readiness for transition assessment. *Int J Adolesc Med Health*. 2015;0(0). doi:10.1515/ijamh-2015-0010

71. Moser A, Stuck AE, Silliman RA, Ganz PA, Clough-Gorr KM. The eight-item modified Medical Outcomes Study Social Support Survey: psychometric evaluation showed excellent performance. *J Clin Epidemiol*. 2012;65(10):1107-1116. doi:10.1016/j.jclinepi.2012.04.007

72. Ferster A, Vermylen C, Cornu G, et al. Hydroxyurea for treatment of severe sickle cell anemia: a pediatric clinical trial. *Blood*. 1996;88(6):1960-1964.

73. Charache S. Fetal hemoglobin, sickling, and sickle cell disease. *Adv Pediatr*. 1990;37:1-31.

74. Platt OS, Thorington BD, Brambilla DJ, et al. Pain in Sickle Cell Disease: Rates and Risk Factors. *N Engl J Med*. 1991;325(1):11-16. doi:10.1056/NEJM199107043250103

75. Odenheimer DJ, Sarnaik SA, Whitten CF, et al. The relationship between fetal hemoglobin and disease severity in children with sickle cell anemia. *Am J Med Genet*. 1987;27(3):525-535. doi:10.1002/ajmg.1320270305

76. Barakat LP, Schwartz LA, Salamon KS, Radcliffe J. A family-based randomized controlled trial of pain intervention for adolescents with sickle cell disease. *J Pediatr Hematol Oncol*. 2010;32(7):540-547. doi:10.1097/MPH.0b013e3181e793f9

77. Logan DE, Radcliffe J, Smith-Whitley K. Parent factors and adolescent sickle cell disease: associations with patterns of health service use. *J Pediatr Psychol*. 2002;27(5):475-484.

78. Bureau UC. Household Pulse Survey. Census.gov. Accessed May 24, 2020. https://www.census.gov/householdpulsedata

79. Bracho-Sanchez DE. Delays in vaccinations, delays in care: How fear of Covid-19 is affecting children's health. CNN. Accessed May 23, 2020.

80. Parmanto, B, Nelson Lewis, A, Graham, KM Development of the Telehealth Usability Questionnaire (TUQ). Int J Telerehabil 2016; 8: 3–10.
