# Supplementary material for: Community Health Worker and Mobile Health Interventions for Quality of Life Among Young Adults With Sickle Cell Disease: A Randomized Clinical Trial
Source: JAMA Netw Open. 2025 Nov 17;8(11):e2543571. doi: 10.1001/jamanetworkopen.2025.43571 (PMC12625686; doi:10.1001/jamanetworkopen.2025.43571)
Supplement: Supplement 2. — eTable. Change in Outcome Scores Between Baseline and First Follow-Up by Randomization Group eAppendix 1. Enhanced Usual Care Checklist (12 Months) eAppendix 2. Enhanced Usual Care Checklist (Social Work) [file jamanetwopen-e2543571-s002.pdf]

Supplementary Online Content

Jan S, Steinway C, Belton T, et al. Community health worker and mobile health interventions for quality of life among young adults with sickle cell disease: a randomized clinical trial. *JAMA Netw Open.* 2025;8(11):e2543571. doi:10.1001/jamanetworkopen.2025.43571

eTable. Change in Outcome Scores Between Baseline and First Follow-Up by Randomization Group
eAppendix 1. Enhanced Usual Care Checklist (12 Months)
eAppendix 2. Enhanced Usual Care Checklist (Social Work)

This supplementary material has been provided by the authors to give readers additional information about their work.

© 2025 Jan S et al. *JAMA Network Open*.

eTable. Change in Outcome Scores Between Baseline and First Follow-Up by Randomization Group

	**EUC**	**CHW + EUC**	**p value**	**iManage SCD + EUC**	**p value**
Overall PedsQL	N=107	N=94		N=104	
Change from baseline to first follow-up Median (IQR)	-2.9<br>(-11, 5.2)	2.3<br>(-4.1, 12.2)	0.001	0<br>(-7.6, 7.8)	0.067
SCD Knowledge (SCDKQ)	N=110	N=97		N=110	
Change from baseline to first follow-up Median (IQR)	0<br>(-1, 1)	0<br>(-1, 1)	0.993	0<br>(-1, 1)	0.124
Transition Readiness (TRAQ Overall)	N=108	N=93		N=104	
Change from baseline to first follow-up Median (IQR)	0.3<br>(0.1, 0.6)	0.2<br>(-0.1, 0.5)	0.117	0.2<br>(0, 0.5)	0.386
Social Support (MOS-SSS Overall)	N=104	N = 92		N=101	
Change from baseline to first follow-up Median (IQR)	0<br>(-7.2, 9.2)	5.3<br>(-5.9, 13.8)	0.117	0<br>(-5.3, 11.8)	0.377

© 2025 Jan S et al. *JAMA Network Open*.

eAppendix 1. Enhanced Usual Care Checklist (12 Months)

Usual Care Medical- 12 Months

Screen ID	
**Baseline date: [date_signed]**	
Ever tested for COVID-19?	◯ Yes<br>◯ No
If yes, ever tested positive?	◯ Yes<br>◯ No
Date positive:	______________
Ever received the COVID-19 antibody test?	◯ Yes<br>◯ No
If yes, ever tested positive?	◯ Yes<br>◯ No
Date positive antibody::	______________
Last Brain MRI	______________
Last TCD	______________
TCD Status	◯ Normal<br>◯ Abnormal
Last neuropsychic eval	______________
Last ophtho eval	______________
Last PFT	______________
Last UA	______________
UA Status	◯ Normal<br>◯ Abnormal
Last ferritin	______________
Ferritin status	◯ Normal<br>◯ Abnormal

© 2025 Jan S et al. *JAMA Network Open*.

Last transfusion	______________________
RBC alloantibodies	◯ Positive<br>◯ Negative<br>◯ N/A
Audiogram if on chelation therapy	______________________
History of Depression or Anxiety	◯ Yes<br>◯ No
History of Intellectual Disability	◯ Yes<br>◯ No
Hemoglobin Level (g/dL)	______________________
Date Measured	______________________
Mean corpuscular volume (fL)	______________________
Date Measured	______________________
Reticulocyte count (%)	______________________
Date Measured	______________________
Fetal hemoglobin percentage	______________________
Date Measured	______________________

Other

Patient interviewed alone	◯ Yes<br>◯ No

© 2025 Jan S et al. *JAMA Network Open*.

Patient can report	Yes	No
their SCD type	○	○
baseline hemoglobin	○	○
baseline oxygen saturation	○	○
how medications and supplies are refilled	○	○
what to do when experiencing side effects	○	○
medications, dosages, side effects	○	○
how to make an appointment	○	○
to call for unusual changes in health	○	○
implications of non-adherence	○	○

Discussed with patient:

Reproductive Health ______________________

Genetic Counseling ______________________

Medical Records transferred to adult provider ______________________

Ideal :

Appointments:

Dental visit in the last year ○ Yes ○ No

Vaccinations:

Meningococcal Vaccine ______________________

Pneumococcal Vaccine ______________________

Flu vaccine ______________________

© 2025 Jan S et al. *JAMA Network Open*.

eAppendix 2. Enhanced Usual Care Checklist (Social Work)

Usual Care SW - 12 Months

**First Name: [pt_first_name]**<br>**Last Name: [pt_last_name]**<br>**Baseline date: [date_signed]**	
1. Emergency planning - who to contact when there is a bad reaction to medications; knows when to contract a doctor for unusual changes in health	◯ Yes<br>◯ No
2. Follows-up on referrals, check-ups, or labs	◯ Yes<br>◯ No
3. Understands how to apply for health insurance if current coverage is lost/ how to recertify health insurance PRIOR to it lapsing	◯ Yes<br>◯ No
4. Knows what health insurance covers	◯ Yes<br>◯ No
5. Knows how to complete medical history forms	◯ Yes<br>◯ No
6. Keeps a calendar or list of medical appointments	◯ Yes<br>◯ No
7. Makes a list of questions before a doctor's visit	◯ Yes<br>◯ No
8. Tells medical providers how they are feeling	◯ Yes<br>◯ No
9. PCP is established prior to transition/has an understanding of how to find adult doctors	◯ Yes<br>◯ No
10. Role playing new ED or health system	◯ Yes<br>◯ No

**Transition: Self-Management**	
The patient has adequately developed self-management skills	◯ Yes<br>◯ No
1. Understanding of medical condition and medications	◯ Yes<br>◯ No
2. Knows how to make an appointment	◯ Yes<br>◯ No
3. Spends time alone with the provider	◯ Yes<br>◯ No

© 2025 Jan S et al. *JAMA Network Open*.

4. Has obtained medical records	◯ Yes<br>◯ No
5. Has an understanding of advanced directives	◯ Yes<br>◯ No
6. Knows how to take medications correctly and on	◯ Yes, their own<br>◯ No
7. Reorders medications before they run out	◯ Yes<br>◯ No
8. Refilling medications and supplies	◯ Yes<br>◯ No

**Transition: School**	
The patient has been prepared for the transition from school to college or work.	◯ Yes<br>◯ No
1. A discussion has been had regarding plans for after HS and appropriate referrals have been made.	◯ Yes<br>◯ No
a. Understands the changes in educational accommodations that occur between high school and college/getting accommodations on file.	◯ Yes<br>◯ No<br>◯ N/A
b. Understands financial aspects of school (applying for FAFSA, etc.)	◯ Yes<br>◯ No<br>◯ N/A
c. Knows skills, interests, and strengths in school or work	◯ Yes<br>◯ No
2. Understanding of FMLA and disability (short term and long term)	◯ Yes<br>◯ No
3. Has been referred to the Office of Vocational Rehabilitation	◯ Yes<br>◯ No<br>◯ N/A
4. Has been referred to the appropriate Development Disabilities office	◯ Yes<br>◯ No<br>◯ N/A
5. Understands the importance of a neuropsych evaluation, if indicated (ideally performed while still in high school)	◯ Yes<br>◯ No

© 2025 Jan S et al. *JAMA Network Open*.

Transition: Understanding	
The patient has an understanding of SCD	◯ Yes<br>◯ No
1. Knows SCD type	◯ Yes<br>◯ No
2. Knows Baseline hemoglobin	◯ Yes<br>◯ No
3. Knows implications of not adhering to medical plan	◯ Yes<br>◯ No
4. Knows causes and symptoms of SCD	◯ Yes<br>◯ No
5. Address outstanding behavioral health needs	◯ Yes<br>◯ No
6. Role playing disclosing disease to potential partner/partner hemeglobinopathy testing	◯ Yes<br>◯ No

Transition: Living Situation	
The patient has been asked about their living situation	◯ Yes<br>◯ No
1. Discussed possible living situations as an adult (needs supports, lives with parents, lives in group home)	◯ Yes<br>◯ No
2. Identify personal care needs (can care for personal needs, unable to provide self-care but can direct others, requires total care) [Yes/No]	◯ Yes<br>◯ No

Transition: Transportation	
The patient has been asked about their transportation options	◯ Yes<br>◯ No
1. Identify transportation needs (self, Medicaid, family, public)/Able to arrange transportation via Medicaid (MAS vs. Logisticare)	◯ Yes<br>◯ No
2. Identify potential barriers to affording transportation (ex., bus fare, parking, etc.)	◯ Yes<br>◯ No
3. Identify what they will need transportation for (ex., school, work, medical appointments, pharmacy, ED)	◯ Yes<br>◯ No

© 2025 Jan S et al. *JAMA Network Open*.

Transition: Financial	
The patient has been educated on the financial aspects of adulthood	◯ Yes<br>◯ No
1. Young adult knows that s/he can work	◯ Yes<br>◯ No
2. Young adult knows the importance and timing for re-determinations for disability	◯ Yes<br>◯ No
3. Young adult knows the max hours that s/he can work and still qualify for	◯ Yes<br>◯ No
4. Can manage a budget	◯ Yes<br>◯ No
5. Can manage banking	◯ Yes<br>◯ No
6. Can pay bills on own	◯ Yes<br>◯ No
7. Can make financial decisions	◯ Yes<br>◯ No
8. Aware of benefits/entitlements/public assistance - eligibility & how to apply (ex: SSI, SSD, TANF, SNAP, WIC, housing, healthcare spending account, etc)	◯ Yes<br>◯ No
9. Understands copays and options for other medical assistance	◯ Yes<br>◯ No
10. Understand and review utility medical protection plans/medical certification/energy assistance	◯ Yes<br>◯ No
Patient has been referred to community support groups or social/recreational activities	◯ Yes<br>◯ No

© 2025 Jan S et al. *JAMA Network Open*.
