# Supplementary material for: Community Health Worker and Mobile Health Interventions for Quality of Life Among Young Adults With Sickle Cell Disease: A Randomized Clinical Trial
Source: JAMA Netw Open. 2025 Nov 17;8(11):e2543571. doi: 10.1001/jamanetworkopen.2025.43571 (PMC12625686; doi:10.1001/jamanetworkopen.2025.43571)
Supplement: Supplement 3. — Data Sharing Statement [file jamanetwopen-e2543571-s003.pdf]

Data Sharing Statement

Jan. Community Health Worker and Mobile Health Interventions for Quality of Life Among Young Adults With Sickle Cell Disease. *JAMA Netw Open*. Published November 17, 2025. doi:10.1001/jamanetworkopen.2025.43571

Data
Additional Information: ClinicalTrials.gov NCT03648710: https://clinicaltrials.gov/study/NCT03648710
Data available: Yes
Data types: Deidentified participant data, Data dictionary
How to access data: Deidentified individual participant data will be made available upon request. Proposals for access should be sent to sjan1@northwell.edu. The data will be shared for the purpose of further research with a signed data use agreement.
When available: With publication

Supporting Documents
Document types: None

Additional Information
Who can access the data: Data will be made available to researchers whose proposed use of the data has been approved.
Types of analyses: Data will be made available for the purposes of additional research.
Mechanisms of data availability: Data will be made available after a signed data access agreement is in place.
